# Destabilization of linker histone H1.2 is essential for ATM activation and DNA damage repair

**DOI:** 10.1038/s41422-018-0048-0

**Published:** 2018-05-29

**Authors:** Zhiming Li, Yinglu Li, Ming Tang, Bin Peng, Xiaopeng Lu, Qiaoyan Yang, Qian Zhu, Tianyun Hou, Meiting Li, Chaohua Liu, Lina Wang, Xingzhi Xu, Ying Zhao, Haiying Wang, Yang Yang, Wei-Guo Zhu

**Affiliations:** 1 Key Laboratory of Carcinogenesis and Translational Research (Ministry of Education), Beijing Key Laboratory of Protein Posttranslational Modifications and Cell Function, Department of Biochemistry and Molecular Biology, School of Basic Medical Sciences, Peking University Health Science Center, Beijing, 100191 China; 2 Guangdong Key Laboratory of Genome Instability and Human Disease Prevention, Department of Biochemistry and Molecular Biology, School of Medicine, Shenzhen University, Shenzhen, 518060 China

## Abstract

Linker histone H1 is a master regulator of higher order chromatin structure, but its involvement in the DNA damage response and repair is unclear. Here, we report that linker histone H1.2 is an essential regulator of ataxia telangiectasia mutated (ATM) activation. We show that H1.2 protects chromatin from aberrant ATM activation through direct interaction with the ATM HEAT repeat domain and inhibition of MRE11-RAD50-NBS1 (MRN) complex-dependent ATM recruitment. Upon DNA damage, H1.2 undergoes rapid PARP1-dependent chromatin dissociation through poly-ADP-ribosylation (PARylation) of its C terminus and further proteasomal degradation. Inhibition of H1.2 displacement by PARP1 depletion or an H1.2 PARylation-dead mutation compromises ATM activation and DNA damage repair, thus leading to impaired cell survival. Taken together, our findings suggest that linker histone H1.2 functions as a physiological barrier for ATM to target the chromatin, and PARylation-mediated active H1.2 turnover is required for robust ATM activation and DNA damage repair.

## Introduction

The nucleosome, as a basic unit of chromatin, is composed of an octamer of core histones associated with about 146 bp of DNA. Linker histone H1 serves as an intranucleosomal architectural protein that unlike the relatively stable organization of core histones, is dynamically bound to chromatin to regulate chromatin accessibility and plasticity.^1,2^ H1 has some 11 isoforms in mammalian cells, which redundantly regulate higher order chromatin structure. Although isoform-specific deletion of H1 has no detectable phenotypes in protozoans or mice,^3,4^ the combined depletion of three isoforms in mouse embryonic stem (ES) cells leads to profound chromatin structural defects.^5^ Deletion of H1 in *Drosophila* leads to high frequency of sister-chromatid exchanges and DNA breaks,^6^ indicating that H1 is a critical regulator of genome stability and integrity.

In addition to its role in controlling chromatin structure, there is accumulating evidence that H1 also participates in the regulation of the DNA damage response and repair, but its precise role remains controversial. In yeast, depletion of H1 up-regulates the homologous recombination (HR) repair machinery and increases resistance to DNA damage.^7^ In addition, mouse ES cells with reduced H1 levels show increased DNA damage signaling and hyper-resistance to DNA-damaging agents.^8^ Others have reported that H1 amplifies ubiquitin signals in the DNA damage response, whereby RNF8 coordinates with RNF168 to promote the recruitment of downstream proteins, thus facilitating DNA repair.^9^ H1 also enhances the backup non-homologous end-joining (NHEJ) pathway by stimulating the activities of DNA ligase IV and III.^10^ Nevertheless, the exact mechanisms underlying the role of H1 in the DNA damage response and repair need to be further elucidated. As one of the most abundant H1 variants, linker histone H1.2 is unique among its family members as it specifically regulates DNA damage-induced apoptosis. Moreover, deletion of H1.2 has been shown to render cancer cells or mice resistant to DNA damaging agents.^11^ In addition, H1.2 shows a distinct preference for AT-rich DNA regions, which tend to be more fragile upon DNA damage due to weaker hydrogen bonds, while other H1 isoforms prefer to bind to GC-rich regions.^12^ These data raise the possibility that H1.2 may have specific roles in regulating the DNA damage response and repair.

Ataxia telangiectasia mutated (ATM) is a master kinase involved in the DNA damage response and repair, which exists as an inactive homodimer or higher order multimer under basal conditions.^13^ Activation of ATM is a complex and tightly regulated process that requires exposure of DNA breaks, a cascade of acetylation and phosphorylation, and the assembly of the MRE11-RAD50-NBS1 (MRN) complex.^13–18^ Numerous cellular processes have been implicated in ATM activation and signaling, including PARP1-mediated poly-ADP-ribosylation (PARylation) during DNA damage.^19^ ATM activation may be associated with structural changes to chromatin as the induction of perturbations to chromatin using sodium chloride (NaCl), chloroquine (CHQ) or histone deacetylase (HDAC) inhibitors can potently activate ATM without eliciting DNA damage.^13^ Chromatin interactions modulated by the nucleosome-binding protein HMGN1 through the regulation of histone acetylation are also essential for ATM activation.^20^ Phosphorylation of TIP60 by c-Abl upon chromatin disruption promotes ATM acetylation and subsequent activation.^21^ Finally, DNA damage-induced displacement of the spliceosome and formation of R-loops activate ATM via a non-canonical pathway.^22^ Together, these reports suggest that ATM activation is indeed regulated by chromatin alterations.

The precise molecular mechanisms that are required to restrain ATM under basal conditions and trigger ATM activation upon DNA damage remain uncertain, but it is reasonable to speculate that ATM may be regulated by chromatin-related factors, such as the linker histone H1. Given that H1 is critical for modulating chromatin dynamics and genome stability, it is possible that H1, or one of its specific isoforms, may be associated with ATM activation. Here, we studied the role of linker histone H1 in the DNA damage response and repair. We report a novel mechanism by which H1.2, but not other H1 isoforms, regulates DNA damage response and repair through the repression of ATM recruitment and activation. Upon DNA damage, H1.2 is rapidly poly-ADP-ribosylated (PARylated) at its C terminus and detaches from chromatin for degradation. Our data reveal a conceptually new functional link between chromatin alterations, H1.2 destabilization and ATM activation.

## Results

### Linker histone H1.2 attenuates the ATM-dependent DNA damage response

**Fig. 1:**
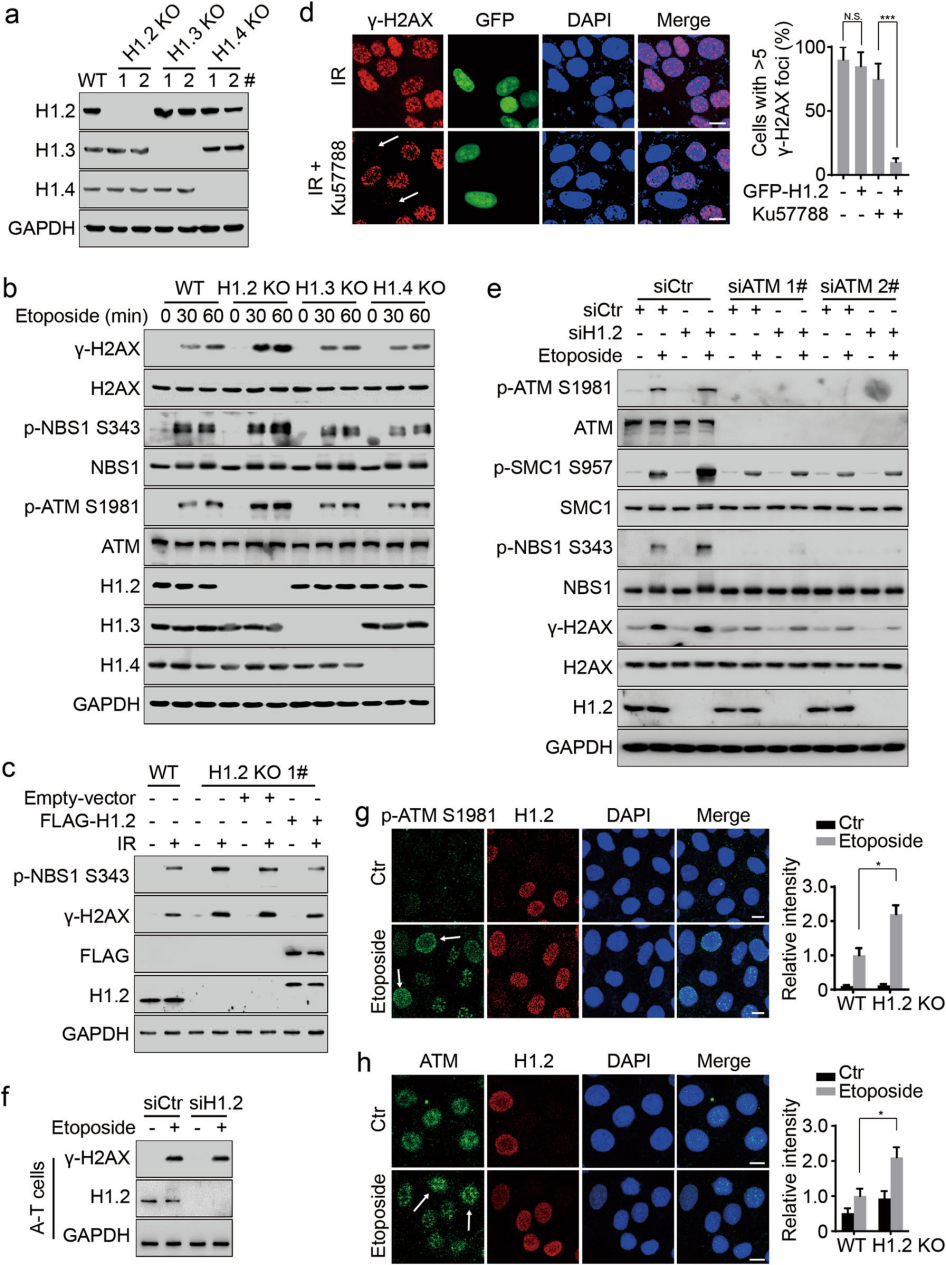
Linker histone H1.2 attenuates the ATM-dependent DNA damage response. **a** Immunoblots for H1.2, H1.3 and H1.4 protein levels in wild-type, H1.2, H1.3 or H1.4 KO HeLa cells. 1# and 2# indicate two clones which were generated using different sgRNAs. **b** Wild-type, H1.2, H1.3 or H1.4 KO (1#) HeLa cells were treated with 40 μM etoposide for 0, 30 and 60 min and analyzed by immunoblotting. **c** Wild-type and H1.2 KO (1#) HeLa cells were transfected with the indicated plasmids with or without exposure to 10 Gy IR and analyzed by immunoblotting 1 h post IR. **d** HeLa cells were transfected with GFP-H1.2 and exposed to 10 Gy irradiation (IR) with or without 2 h prior exposure to 2 μM Ku57788. Cells were collected 1 h post IR and subjected to immunofluorescent assay. Cells with >5 γ-H2AX foci were counted. The data represent the mean ± SD. Scale bars, 10 μm. **e** HeLa cells were transfected with the indicated siRNAs and treated with 40 μM etoposide for 2 h and analyzed by immunoblotting. **f** A-T cells were transfected with the indicated siRNAs and treated with 40 μM etoposide for 1 h and analyzed by immunoblotting. **g**, **h** Wild type and H1.2 KO (1#) HeLa cells were mixed and then treated with 40 μM etoposide for 2 h or left untreated (Ctr) and analyzed by immunofluorescence. The intensity of ATM or phospho-ATM S1981 in the etoposide-treated wild-type cells was normalized to 1. The arrows indicate representative cells. All data represent the mean ± SD. Scale bars, 10 μm

To explore the potential connections between linker histone H1 and ATM, we generated H1.2, H1.3 and H1.4 variant-specific knockout (KO) HeLa cells using **c**lustered **r**egularly **i**nterspaced **s**hort **p**alindromic **r**epeats (CRISPR)-Cas9 technology (Fig. 1a). Notably, etoposide or ionizing radiation (IR)-induced phosphorylation of H2AX, NBS1, SMC1 and ATM was markedly elevated in H1.2 KO cells, but not in H1.3 or H1.4 KO cells (Fig. 1b, c; Supplementary information, Figure S1a). The formation of γ-H2AX foci upon DNA damage was also clearly promoted in H1.2 KO cells (Supplementary information, Figure S1b). Rescue experiments, we performed by transfecting H1.2 into H1.2 KO cells, showed that reintroduction of H1.2 could potently suppress the elevated ATM activation (Fig. 1c; Supplementary information, Figure S1c). Interestingly, PARylation, another chromatin modification in the initial response to DNA damage, was not altered when H1.2 was depleted (Supplementary information, Figure S1d). Moreover, the ultraviolet (UV)-induced DNA damage response was not influenced by H1.2 depletion (Supplementary information, Figure S1e), suggesting that H1.2 suppresses DNA double-strand break (DSB)-induced phosphorylation signaling.

To determine which kinase is involved in phosphorylation signaling opposed by H1.2, we pre-treated cells with specific inhibitors against ATM (Ku55933) or DNA-dependent protein kinase (DNA-PK) (Ku57788), as these two kinases have a known involvement in DSB-induced phosphorylation.^23^ Following DNA damage, H1.2 depletion-enhanced phosphorylation was abrogated in Ku55933-treated cells, but not Ku57788-treated cells (Supplementary information, Figure S1f). In addition, direct over-expression of H1.2 did not lead to significant inhibition of γ-H2AX foci formation (Fig. 1d), indicating that H1.2 may only interfere with one of the redundant DSB-responding kinases. This accords with previous reports that inhibition of either ATM or DNA-PK alone showed limited effects on IR-induced γ-H2AX.^24^ However, H1.2 over-expression led to markedly reduced γ-H2AX levels when DNA-PK was inhibited by Ku57788 treatment (Fig. 1d; Supplementary information, Figure S1g), suggesting that H1.2 specifically inhibits ATM, but not DNA-PK activity. Similarly, the enhanced phosphorylation of ATM or its substrates resulting from H1.2 depletion was evidently prevented by ATM knockdown, but not by knockdown of ATR or DNA-PKcs (Fig. 1e; Supplementary information, Figure S1h and i). Moreover, H1.2 knockdown in ATM-deficient A-T cells had no significant influence on γ-H2AX levels (Fig. 1f), suggesting a direct role of ATM in H1.2-opposed phosphorylation signaling. Immunofluorescent staining after in situ detergent extraction also showed that H1.2 KO led to increased ATM activation and recruitment to chromatin upon DNA damage (Fig. 1g, h). Finally, although H1.2 depletion led to a mild cell cycle arrest in G1 phase, DNA damage-induced ATM activation was still increased upon H1.2 deletion when cells were synchronized using a double thymidine block (Supplementary information, Figure S1j and k), suggesting that regulation of ATM by H1.2 is independent of the cell cycle phase. These data indicate that H1.2 attenuates the ATM-dependent DNA damage response in vivo and protects chromatin from abnormal ATM recruitment and activation.

### Linker histone H1.2 interacts with ATM and directly inhibits its activity

**Fig. 2:**
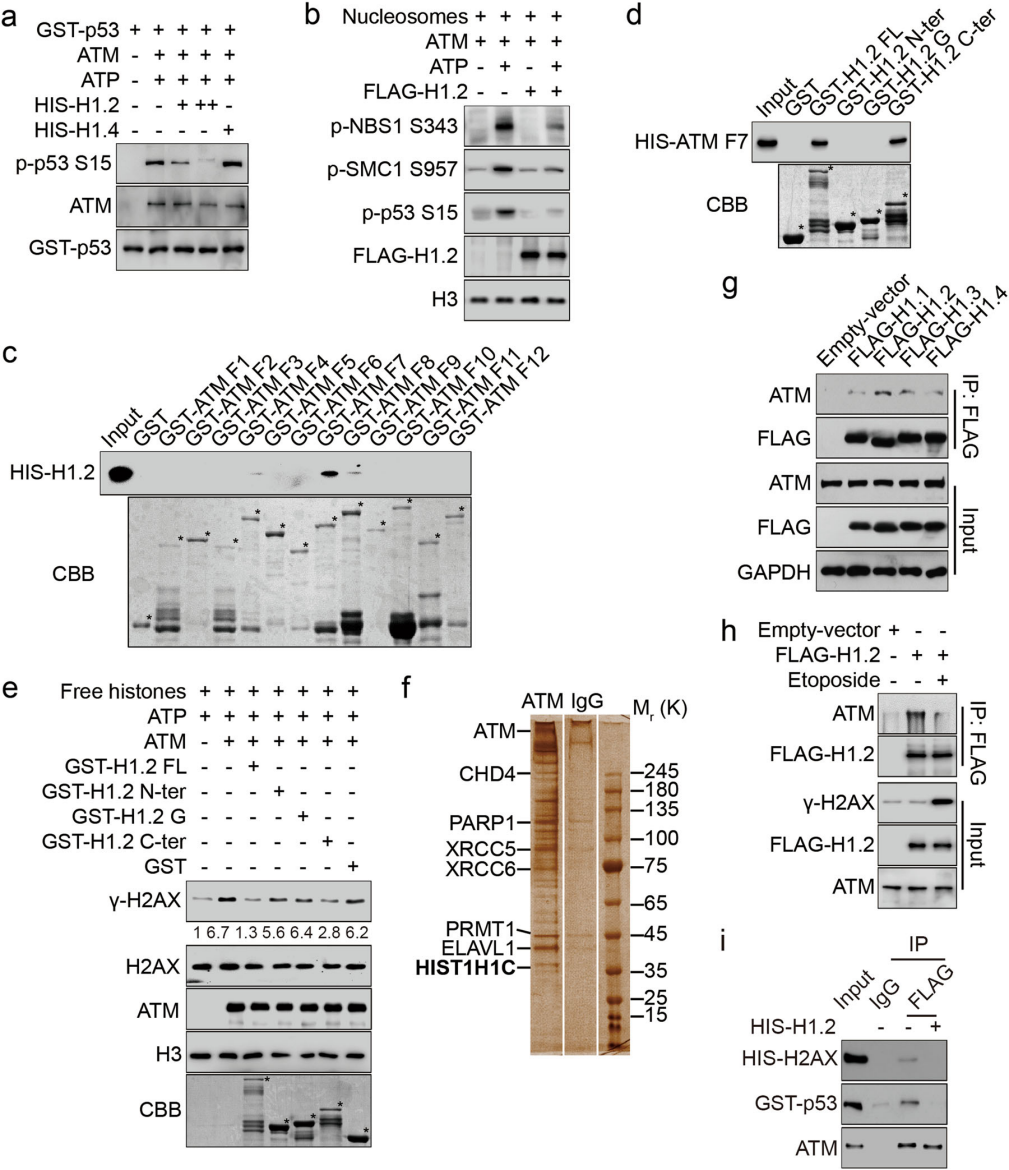
Linker histone H1.2 interacts with ATM and directly inhibits its activity. **a** An N-terminal GST-p53 (1–99 aa) peptide was used as a substrate for in vitro phosphorylation assay with or without HIS-H1.2/H1.4. **b** HCT116 cells were transfected with FLAG-H1.2 or an empty vector and mononucleosomes were extracted and subjected to in vitro phosphorylation. **c** GST alone or GST-ATM fragments were incubated with HIS-H1.2 for the GST pull-down assay. * indicates specific protein bands. **d** GST alone or GST-H1.2 fragments were incubated with HIS-ATM fragment 7 (F7, 1239–1770 aa) for GST pull-down assay. * indicates specific protein bands. **e** Free histones extracted from HeLa cells were used as substrates for in vitro phosphorylation in the presence of GST-H1.2 fragments or GST alone. The relative intensity of γ-H2AX/H2AX was calculated. * indicates specific protein bands. **f** Total HeLa cell lysates were immunoprecipitated with anti-ATM or anti-IgG antibodies. The precipitated proteins were analyzed by mass spectrometry after SDS-PAGE electrophoresis and silver staining. Name in bold indicates the desired protein. **g** HEK293T cells were transfected with the indicated plasmids and subjected to Co-IP assay with FLAG-conjugated M2 beads. **h** HeLa cells were transfected with FLAG-H1.2 or an empty vector and treated as indicated with 40 μM etoposide for 2 h. Total cell lysates were immunoprecipitated using FLAG-conjugated M2 beads and analyzed by immunoblotting. **i** ATM was immunoprecipitated using FLAG-conjugated M2 beads in HEK293T cells overexpressed with FLAG-ATM and incubated with (−) or without (+) recombinant HIS-H1.2 in kinase buffer. Recombinant ATM substrates, including HIS-H2AX (full-length) and GST-p53 (amino acids 1–99) were incubated without ATP. The interacting proteins were eluted and analyzed by immunoblotting

To test the possibility that H1.2 directly regulates ATM activity, we established an in vitro kinase assay whereby purified ATM protein was incubated with different ATM substrates, including an N-terminal glutathione S-transferase (GST)-p53 (1–99 aa) peptide, free histones and mononucleosomes. ATM activity was markedly repressed when recombinant H1.2, but not H1.4, was introduced, as measured by the levels of p53 phosphorylation on serine 15 (S15) and γ-H2AX (Fig. 2a; Supplementary information, Figure S2a and b). ATR kinase activity, however, was not affected by H1.2 (Supplementary information, Figure S2c). In addition, ATM exhibited lower activity towards mononucleosomes when H1.2 was over-expressed, as determined by the phosphorylation levels of ATM chromatin substrates (Fig. 2b; Supplementary information, Figure S2d). Together, these findings suggest that H1.2 directly inhibits ATM activity in vitro.

We next used a GST pull-down assay with purified fragments of GST-ATM and HIS-H1.2 to determine whether H1.2 directly interacts with ATM. We found that fragment 7 (F7) containing a specific region of ATM HEAT repeat domain (1239–1770 aa) directly interacted with H1.2 (Fig. 2c; Supplementary information, Figure S2e). GST-fragments of H1.2 were also purified and incubated with HIS-ATM F7 and the C-terminal domain of H1.2 (113–213 aa) was identified to be responsible for its binding to ATM (Fig. 2d; Supplementary information, Figure S2f). Accordingly, introduction of the H1.2 C-terminal domain attenuated ATM activity in vitro (Fig. 2e). In addition, mass spectrometric analysis of interacting proteins detected H1.2 as co-purifying with ATM, which was confirmed by subsequent co-immunoprecipitation (Co-IP) (Fig. 2f; Supplementary information, Figure S2g and h). Moreover, we showed that interaction between ATM and H1.2 was specific, as other H1 variants exhibited a much weaker binding affinity to ATM and the interacting domain was also different (Fig. 2g; Supplementary information, Figure S2i). Upon etoposide-induced DNA damage, the interaction between ATM and H1.2 was markedly decreased (Fig. 2h). The fact that the ATM HEAT repeat domain is critical for binding of its substrates prompted us to examine whether H1.2 interferes with ATM binding to other partners. Interestingly, we found that H1.2 reduced the binding of ATM to substrates that included H2AX and p53 (Fig. 2i). Taken together, these findings confirm that H1.2 inhibits ATM activity via direct binding with the ATM HEAT repeat domain.

### Linker histone H1.2 inhibits ATM recruitment and activation by interacting with MRN

**Fig. 3:**
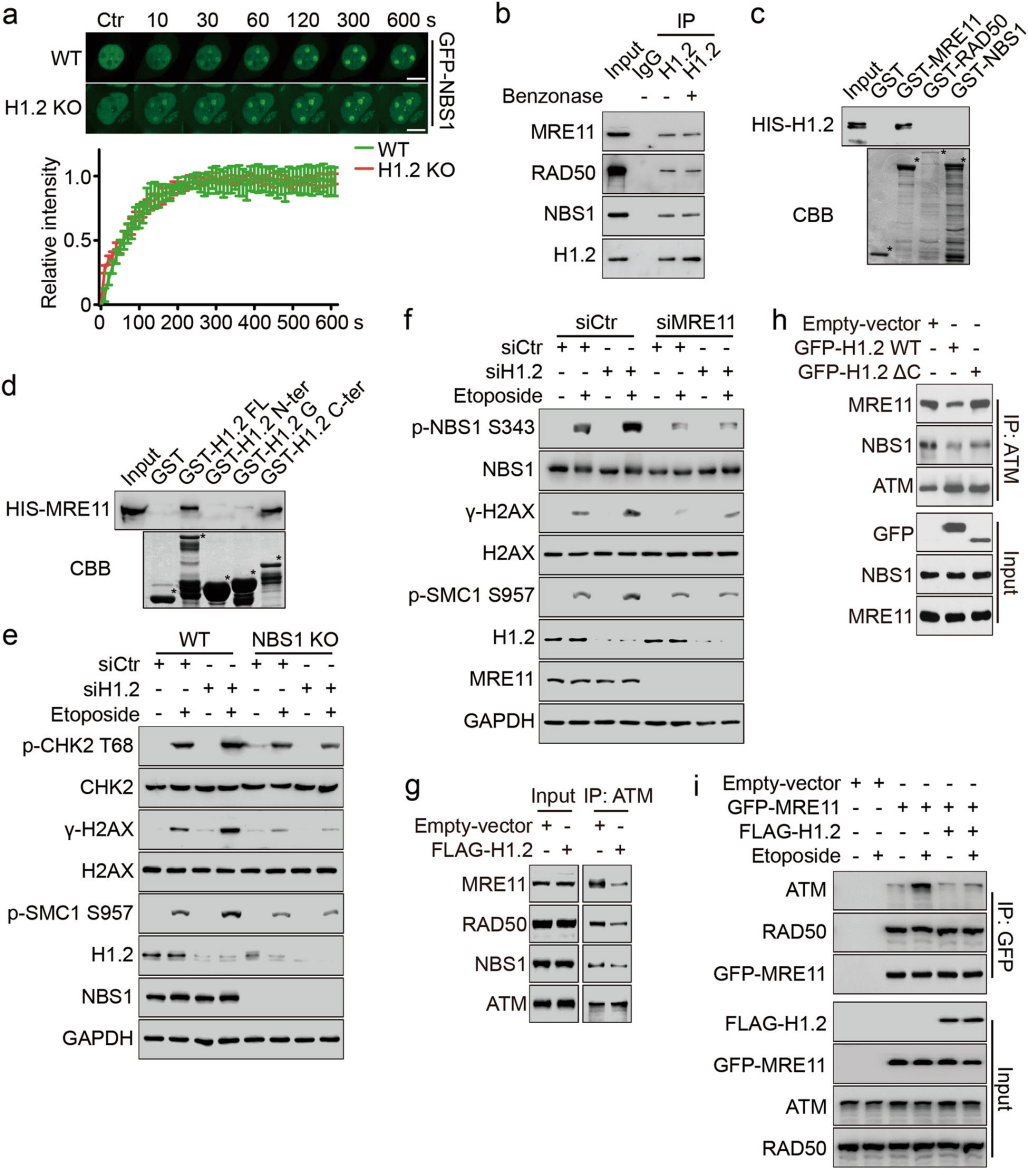
Linker histone H1.2 inhibits ATM recruitment and activation by interacting with MRN. **a** Wild type and H1.2 KO (1#) HeLa cells were transfected with GFP-NBS1 and subjected to laser micro-irradiation-coupled live-cell imaging. Images were taken every 10 s for 10 min and the relative intensity of the irradiation path signal was shown. The data represent the mean ± SD. Scale bars, 10 μm. **b** HeLa cells extracts were analyzed by Co-IP assay with or without benzonase treatment with the indicated antibodies. **c** GST alone or GST-MRE11, RAD50 and NBS1 were incubated with HIS-H1.2 for GST pull-down assay. * indicates specific protein bands. **d** GST alone or GST-H1.2 fragments were incubated with HIS-MRE11 for GST pull-down assay. * indicates specific protein bands. **e** Wild type or NBS1 KO HeLa cells were transfected with the indicated siRNAs and treated with 40 μM etoposide for 2 h and analyzed by immunoblotting. **f** HeLa cells were transfected with the indicated siRNAs and treated with 40 μM etoposide for 2 h and analyzed by immunoblotting. **g**, **h** HeLa cells were transfected with the indicated plasmids, and the whole cell lysates were immunoprecipitated with ATM antibody and analyzed by immunoblotting. **i** HeLa cells were transfected with the indicated plasmids and treated with 40 μM etoposide for 2 h. Whole cell extracts were prepared and analyzed by Co-IP assay and immunoblotting with the indicated antibodies

MRN is known to be essential for the recruitment of ATM in DNA damage response.^25^ To clarify how H1.2 interferes with ATM recruitment, we therefore examined the relationship between H1.2 and the MRN complex. We found that H1.2 KO had little effect on the initial recruitment of the MRN complex, as monitored by live-cell imaging of GFP-NBS1 and GFP-MRE11 localization following laser micro-irradiation (Fig. 3a; Supplementary information, Figure S3a), suggesting that H1.2 may function downstream of MRN. We obtained similar results by chromatin fractionation and confocal microscopic analysis of MRN complex components after etoposide treatment (Supplementary information, Figure S3b and c). Further explorations of the interactions between the MRN complex and H1.2 by Co-IP showed that H1.2 interacted with the MRN complex (Fig. 3b; Supplementary information, Figure S3d). In addition, treatment with benzonase, a nuclease which cleaves multiple forms of DNA, did not alter the interaction (Fig. 3b), indicating that H1.2 interacts with MRN in a DNA-independent manner. Specifically, in vitro GST pull-down assays identified that the C-terminal domain of H1.2 directly interacted with MRE11, but not RAD50 or NBS1 (Fig. 3c, d; Supplementary information, Figure S3e). Similarly, the in vitro interaction was also not affected by benzonase (Supplementary information, Figure S3f), further suggesting that H1.2 directly interacts with MRE11.

To further determine whether H1.2 attenuates ATM recruitment and activation through MRN, we performed H1.2 knockdown in NBS1 KO or MRE11 knockdown cells. It was shown that deletion of NBS1 or MRE11 impaired ATM activation, whereas H1.2 depletion failed to rescue this repression (Fig. 3e, f), suggesting that both recruitment of MRN and release of H1.2 are required for ATM activation. In addition, the interaction between the MRN complex and ATM was attenuated by the over-expression of H1.2 even without DNA damage (Fig. 3g; Supplementary information, Figure S3g). Moreover, C terminally deleted (ΔC) H1.2, which could not interact with ATM or MRE11, failed to do so (Fig. 3h), indicating that H1.2 and MRN may compete for the binding of ATM. Upon etoposide treatment, the interaction between ATM and the MRN complex was enhanced, which could again be suppressed by over-expression of H1.2 (Fig. 3i; Supplementary information, Figure S3h). We could also show that over-expression of H1.2 did not alter the interaction between the MRN components (Fig. 3i; Supplementary information, Figure S3g and h). Together, these results suggest that H1.2 inhibits ATM recruitment and subsequent activation by sequestering its binding to the MRN complex.

### Linker histone H1.2 is rapidly displaced and degraded upon DNA damage

**Fig. 4:**
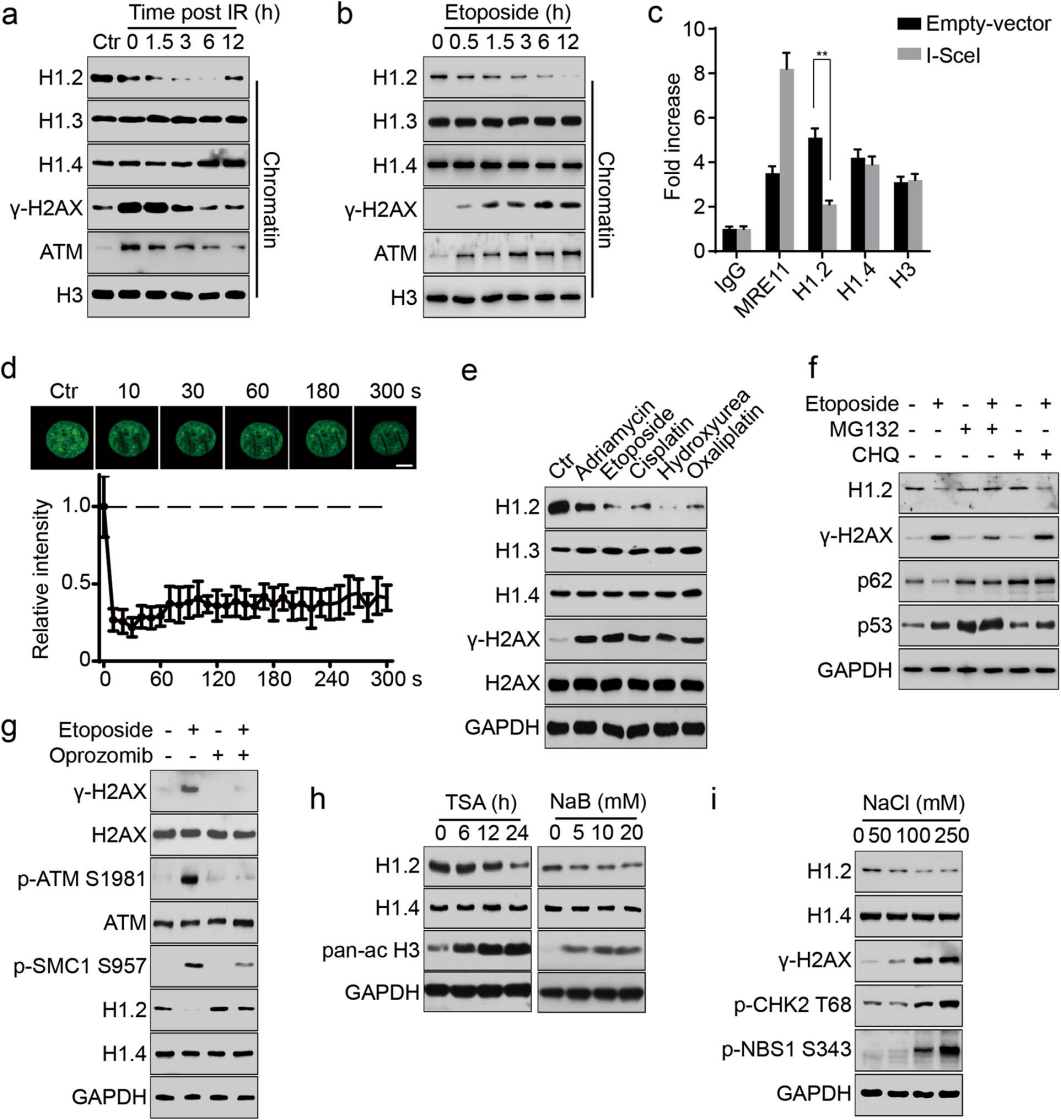
Linker histone H1.2 is rapidly displaced from chromatin and degraded upon DNA damage. **a**, **b** HeLa cells were exposed to 10 Gy irradiation (IR) and released at the indicated time or treated with 20 μM etoposide for the indicated time. Chromatin was fractionated and subjected to immunoblotting. **c** DR-GFP U2OS cells were transfected with I-*Sce*I endonuclease or an empty vector and subjected to chromatin immunoprecipitation with the indicated antibodies followed by real-time PCR 48 h post transfection. All data represent the mean ± SD. **d** HeLa cells were transfected with GFP-H1.2 and subjected to laser micro-irradiation-coupled live-cell imaging. The initial signal intensity of GFP-H1.2 was normalized to 1 and ~15 IR paths from 10 separate cells were calculated. All data represent the mean ± SD. Scale bars, 10 μm. **e** HeLa cells were treated with either 1 μM adriamycin at 1 μM, 20 μM etoposide, 10 μM cisplatin, 2 mM hydroxyurea or 10 μM oxaliplatin for 12 h and analyzed by immunoblotting. **f** HeLa cells were treated with 1 μM MG132 or 50 μM CHQ for 12 h with or without 20 μM etoposide and analyzed by immunoblotting. **g** HeLa cells were treated with etoposide at 20 μM for 12 h with or without Oprozomib at 100 nM and analyzed by immunoblotting. **h** HeLa cells were treated with 1 μM TSA for the indicated time or to increasing concentrations of sodium butyrate (NaB) for 12 h and analyzed by immunoblotting. A pan-ac H3 antibodies was used as a positive control. **i** HeLa cells were exposed to increasing concentrations of sodium chloride (NaCl) for 2 h and analyzed by immunoblotting

To better understand how H1.2 regulates ATM activity under physiological conditions, we examined the dynamics of linker histone in response to DNA damage. Interestingly, after exposing cells to etoposide or IR and analyzing the chromatin fractions, we found that linker histone H1.2, but not other H1 isoforms, was displaced from chromatin, correlating with recruitment and activation of ATM (Fig. 4a, b; Supplementary information, Figure S4a). At later stages of DNA damage repair, H1.2 was gradually restored when chromatin ATM was restored to its basal level (Fig. 4a), which may be associated with completion of the repair process. A chromatin immunoprecipitation (ChIP) assay in DR-GFP U2OS cells also showed that H1.2 dissociated from chromatin upon I-*Sce*I-induced DNA damage whereas H1.4 levels showed no detectable changes at the I-*Sce*I damage site (Fig. 4c). Further experiments using laser micro-irradiation coupled live-cell imaging of GFP-H1.2 localization demonstrated that the dissociation of H1.2 took place immediately (<10 s) after DNA damage was elicited (Fig. 4d). Staining to reveal endogenous H1.2 after laser micro-irradiation also confirmed that H1.2 was rapidly displaced in the irradiation path (Supplementary information, Figure S4b).

To investigate whether H1.2 is destabilized after DNA damage, we treated cells with different DNA damaging stimuli and then assessed H1.2 expression. H1.2 protein levels specifically decreased in response to various DNA damage treatments in different cell lines, whereas the levels of H1.3 and H1.4 were unaltered (Fig. 4e; Supplementary information, Figure S4c). The mRNA levels of H1.2 were not obviously decreased, indicating that the decrease of H1.2 was likely due to protein degradation (Supplementary information, Figure S4d). In addition, DNA damage-induced degradation of H1.2 was both time-dependent and dose-dependent (Supplementary information, Figure S4e). Moreover, H1.2 degradation was not induced by UV, which mainly causes single strand breaks (SSBs) (Supplementary information, Figure S4f). Furthermore, we treated cells with a proteasome inhibitor (MG132) or a lysosome inhibitor (chloroquine, CHQ) to determine which pathway might be responsible for H1.2 degradation. As shown in Fig. 4f, MG132 markedly blocked H1.2 degradation, suggesting that H1.2 was degraded in a proteasome-dependent pathway. An in vitro degradation assay consistently showed that H1.2, but not H1.4, was degraded by 20S proteasomes (Supplementary information, Figure S4g). The degradation was further confirmed to be a ubiquitin-independent process in the cytoplasm (Supplementary information, Figure S4h and i). Interestingly, H1.2 degradation was markedly blocked when treated with a specific 20S proteasome inhibitor (Oprozomib), whereas ATM activation was also evidently inhibited (Fig. 4g), suggesting a positive feedback loop between H1.2 destabilization and ATM activation.

To further confirm the connection between H1.2 destabilization and ATM activation, we next treated cells with either NaCl or HDAC inhibitors (trichostatin A [TSA] and sodium butyrate [NaB]), which are known to activate ATM without causing DNA damage.^13^ Surprisingly, TSA and NaB could induce H1.2 degradation in a time-dependent and dose-dependent manner, respectively (Fig. 4h). H1.2 was also degraded following sodium chloride treatment, and this was accompanied by activation of ATM (Fig. 4i). Together, these results suggest a correlation between chromatin alterations, H1.2 destabilization and ATM activation.

### H1.2 PARylation permits its chromatin displacement upon DNA damage

**Fig. 5:**
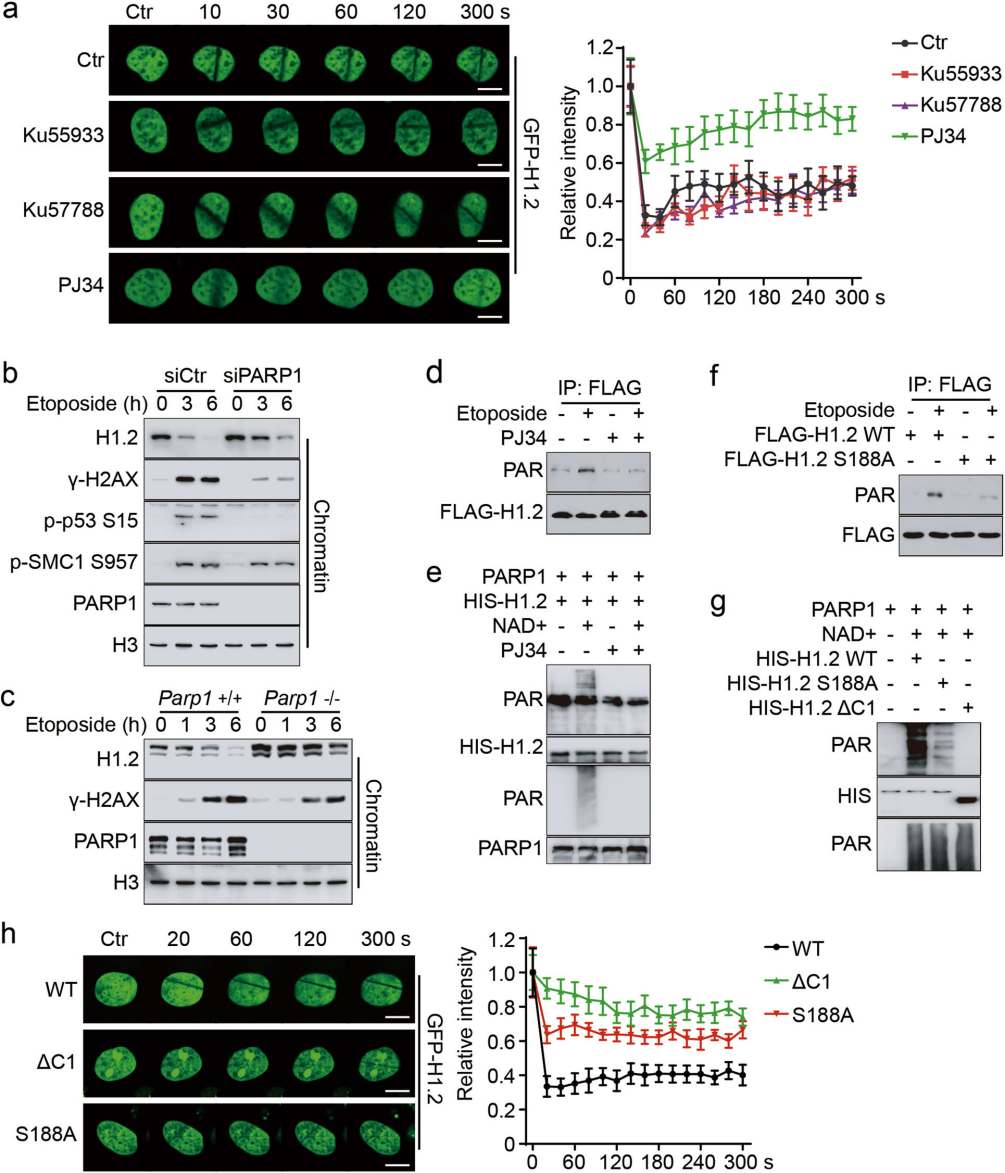
H1.2 PARylation permits its displacement from chromatin upon DNA damage. **a** HeLa cells were transfected with GFP-H1.2 and treated with 20 μM Ku55933 or 2 μM Ku57788 for 4 h or 5 μM PJ34 for 1 h followed by laser micro-irradiation. Images were taken every 10 s for 5 min and quantifications of the IR path signal intensity were shown and ~15 IR paths from 10 separate cells were calculated. The data represent the mean ± SD. Scale bars, 10 μm. **b** HeLa cells were transfected with the indicated siRNAs and treated with 40 μM etoposide for the indicated time. Chromatin was fractionated and analyzed by immunoblotting. **c** *Parp1* wild-type (+/+) or KO (−/−) MEFs were treated with 40 μM etoposide for the indicated time and chromatin was fractionated and analyzed by immunoblotting. **d** HeLa cells were transfected with FLAG-H1.2 and treated with 40 μM etoposide for 15 min with or without 5 μM PJ34 for 1 h. Cell extracts were immunoprecipitated with FLAG-conjugated M2 beads. **e** Recombinant HIS-H1.2 was subjected to in vitro PARylation assay in the presence of NAD^+^ or 10 μM PJ34, as indicated. **f** HeLa cells were transfected with wild-type or S188A mutated FLAG-H1.2 and treated with 40 μM etoposide for 15 min with or without 5 μM PJ34 for 1 h, as indicated. Cells were extracted and immunoprecipitated with FLAG-conjugated M2 beads. **g** Recombinant wild-type, S188A mutated or C1-deleted (ΔC1) HIS-H1.2 were subjected to in vitro PARylation assay. **h** HeLa cells were transfected with wild-type, ΔC1 or S188A mutated GFP-H1.2 and subjected to laser micro-irradiation. Images were taken every 20 s for 5 min and representative images were shown. Quantifications were calculated as in **a**. The data represent the mean ± SD. Scale bars, 10 μm

Previous data supports the notion that H1 dynamics are tightly regulated by its post-translational modifications (PTMs).^26^ We therefore decided to address the mechanisms of DNA damage-induced H1.2 dynamics. Both live-cell imaging and staining of endogenous H1.2 indicated that a specific PARP inhibitor (PJ34), but not ATM or DNA-PKcs inhibitors, significantly repressed the dissociation of H1.2 from chromatin upon laser micro-irradiation (Fig. 5a; Supplementary information, Figure S5a). Mutation of known or putative H1.2 phosphorylation sites that may affect H1’s chromatin binding affinity resulted in only minimal effects on H1.2 displacement (Supplementary information, Figure S5b). Similarly, DNA damage-induced H1.2 dissociation was evidently delayed in PARP1 knockdown HeLa cells and *Parp1* KO (*Parp1*^−/−^) mouse embryonic fibroblasts (MEFs), as confirmed by chromatin fractionation (Fig. 5b, c). As expected, PJ34 inhibited the DNA damage-induced degradation of H1.2 (Supplementary information, Figure S5c).

We then examined how the DNA damage-induced H1.2 dynamics are regulated by PARP1, which is known to catalyze histone PARylation in the DNA damage response.^27^ We detected PARylation of H1.2 upon DNA damage, which was suppressed in the presence of PJ34 (Fig. 5d). In addition, HIS-H1.2 was PARylated when incubated with recombinant human PARP1, activated DNA and NAD^+^, and this effect was repressed when PJ34 was added to the in vitro reaction (Fig. 5e; Supplementary information, Figure S5d). Moreover, we analyzed H1.2 fragments to map the possible PARylation site(s) and found that deletion of the H1.2 C-terminal domain (ΔC) or a short extreme C terminus (ΔC1) largely abrogated PARP1-mediated PARylation (Supplementary information, Figure S5e and f). Previous reports and bioinformatic analyses^28^ together predict that serine 188 of H1.2 is a major PARylation site. Our in vivo and in vitro experiments demonstrated that mutation of this site (S188A) markedly diminished DNA damage-induced and PARP1-mediated PARylation of H1.2 (Fig. 5f, g). Live-cell imaging of the signal intensity across a laser micro-irradiation path also illustrated that the S188A mutation delayed DNA damage-induced H1.2 displacement from chromatin (Fig. 5h). Notably, the ΔC1 mutant displayed an even more significant delay in H1.2 displacement than S188A (Fig. 5h), suggesting that additional sites may be involved in regulating H1.2 dynamics. Furthermore, cycloheximide (CHX) chase experiments showed that the H1.2 S188A mutant was more stable than wild-type H1.2 and etoposide-induced H1.2 degradation was also markedly blocked when S188 was mutated (Supplementary information, Figure S5g and h). Together, these results indicate that H1.2 is dynamically regulated via PARP1-mediated PARylation of its C terminus.

### PARylation of linker histone H1.2 is essential for DNA damage-induced ATM activation

**Fig. 6:**
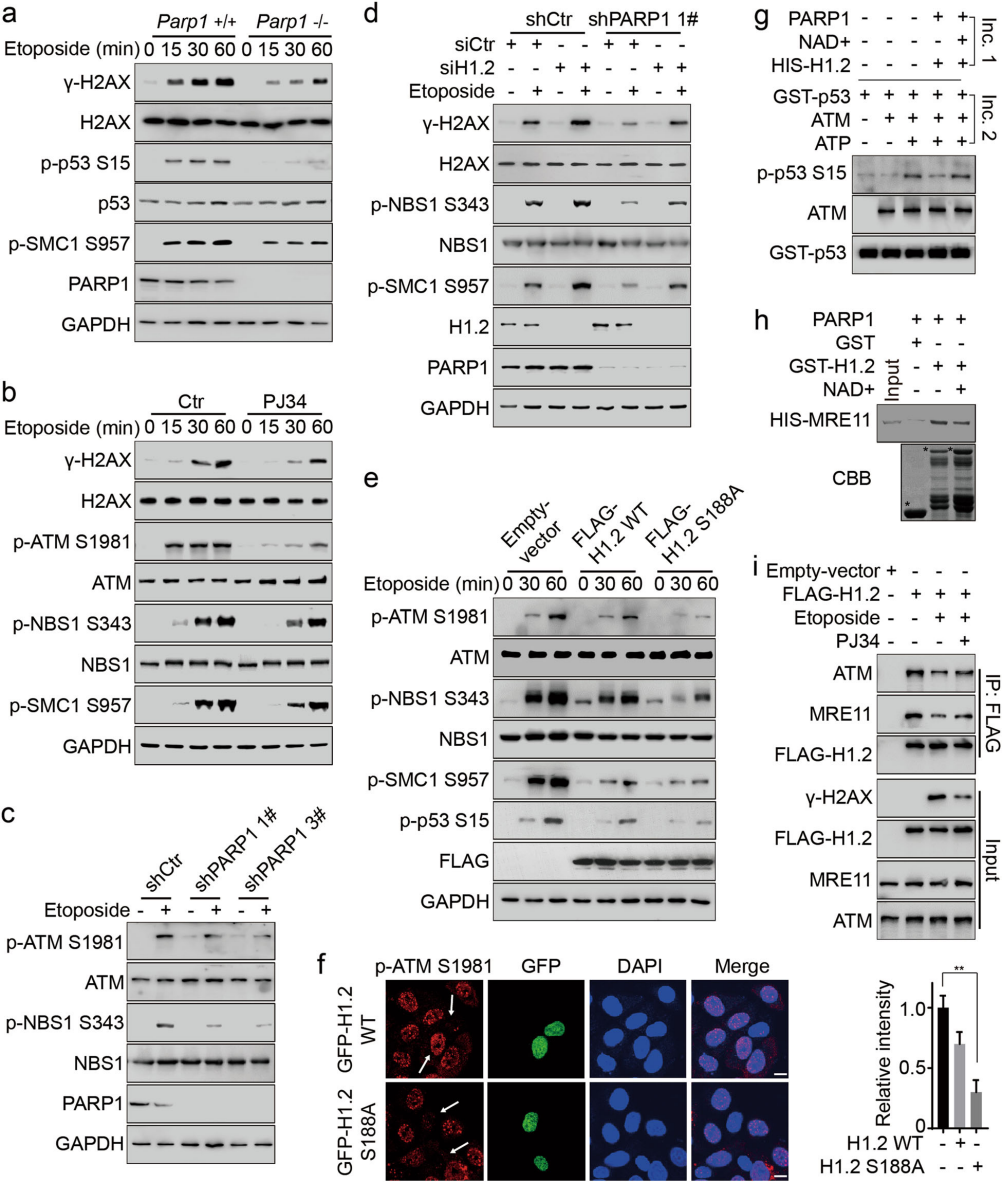
PARylation of H1.2 is essential for ATM activation. **a** *Parp1* wild-type (+/+) or KO (−/−) MEFs were treated with 40 μM etoposide for the indicated time and analyzed by immunoblotting. **b** HeLa cells were treated with 40 μM etoposide for the indicated time with or without exposure to 5 μM PJ34 1 h before etoposide treatment and analyzed by immunoblotting. **c** Two clones of PARP1 stable knockdown (shPARP1 #1 and #3) and control (shCtr) HeLa cells were treated with 40 μM etoposide for 30 min and analyzed by immunoblotting. **d** shPARP1 (1#) and shCtr HeLa cells were transfected with the indicated siRNAs and treated with 40 μM etoposide for 30 min and analyzed by immunoblotting. **e** HCT116 cells were transfected with the indicated plasmids and treated with 40 μM etoposide for the indicated times and analyzed by immunoblotting. **f** HeLa cells were transfected with wild-type or S188A mutated GFP-H1.2, treated with 40 μM etoposide for 2 h and the fluorescence intensity of phospho-ATM S1981 in the untransfected cells was normalized to 1. The arrows indicate representative cells. The data represent the mean ± SD. Scale bars, 10 μm. **g** Recombinant HIS-H1.2 was incubated for 30 min at 37 °C with PARP1 with or without NAD^+^ for in vitro PARylation assay (Incubation 1, Inc. 1). H1.2 was eluted and used for in vitro phosphorylation assay (Incubation 2, Inc. 2). An N-terminal GST-p53 (1–99 aa) peptide was used as the substrate. **h** Recombinant GST-H1.2 was incubated with PARP1 with or without NAD^+^ for in vitro PARylation assay. GST alone and PARylated GST-H1.2 were then incubated with HIS-MRE11 for GST-pulldown assay. * indicates specific protein bands. **i** HeLa cells were transfected with the indicated plasmids and treated with 40 μM etoposide for 1 h or 5 μM PJ34 for 1 h. Whole cell extractions were prepared and subjected to Co-IP assay with FLAG-conjugated M2 beads

ATM activation in *Parp1*^−/−^ MEFs was attenuated following etoposide treatment, consistent with an inhibitory role for H1.2 in regulating ATM (Fig. 6a). Accordingly, inhibition (with PJ34) or knockdown of PARP1 (by stably expressed shRNA) led to compromised ATM activation (Fig. 6b, c; Supplementary information, Figure S5i). This inhibition of ATM activation by PARP1 knockdown or inhibition was restored by H1.2 knockdown (Fig. 6d; Supplementary information, Figure S5j). In addition, over-expression of H1.2 S188A repressed ATM activation (Fig. 6e). Consistently, positive immunofluorescent staining of p-ATM S1981 was not detected in the majority of cells over-expressing H1.2 S188A (Fig. 6f), further supporting the notion that PARP1 regulates ATM activation through PARylation and displacement of H1.2.

To investigate the direct role of H1.2 PARylation in regulating ATM activity, we established a two-step in vitro experiment: recombinant H1.2 was first PARylated in vitro (Incubation 1, Inc. 1). The PARylated H1.2 was then added to an in vitro ATM phosphorylation system in which an N-terminal GST-p53 (1–99 aa) peptide was used as a substrate (Incubation 2, Inc. 2). By measuring the levels of p53 S15 phosphorylation, we found that unmodified H1.2 potently inhibited ATM activity, whereas PARylated H1.2 exhibited weaker effects in repressing ATM activation (Fig. 6g). Similarly, PARylated H1.2 showed decreased binding to MRE11, as demonstrated by an in vitro pull-down assay using differentially PARylated H1.2 (Fig. 6h). In addition, inhibition of PARP1 by PJ34 restored DNA damage-induced reduction of the interaction between H1.2 and ATM or MRE11 (Fig. 6i). Together, these data suggest that PARylation of H1.2 is required for ATM activation in response to DNA damage.

### Linker histone H1.2 dissociation and destabilization are required for DNA repair and cell survival

**Fig. 7:**
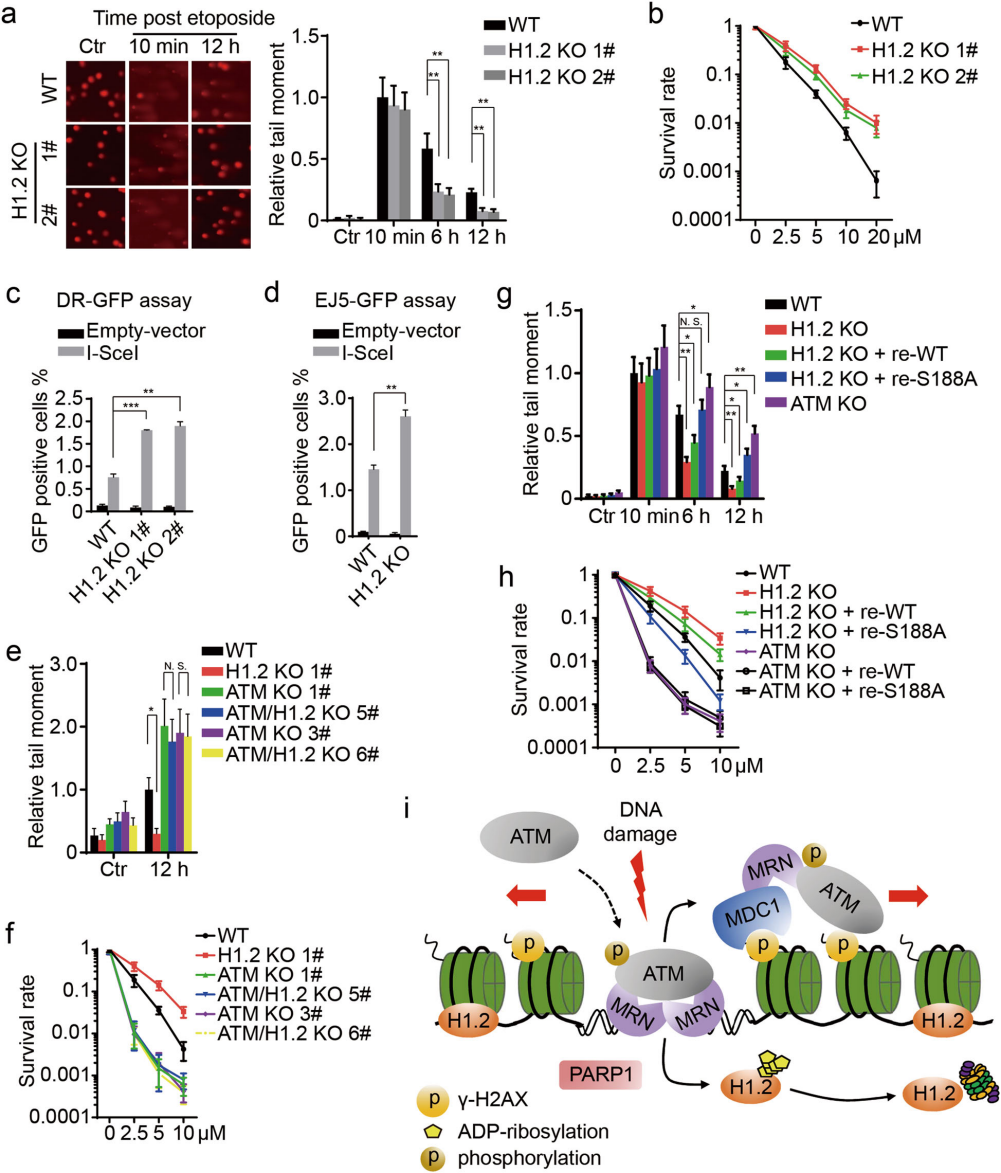
Linker histone H1.2 dissociation and destabilization are required for DNA repair and cell survival. **a**, **b** Wild-type and H1.2 KO (1# and 2#) HeLa cells were analyzed by comet and colony formation assays. The tail moment of wild-type cells at 10 min post treatment was normalized to 1. The data represent the mean ± SD. **c** Wild-type and H1.2 KO (1# and 2#) DR-GFP U2OS cells were analyzed by DR-GFP assay. The data represent the mean ± SD. **d** Wild-type and H1.2 KO pEJ5-GFP U2OS cells were analyzed by EJ5-GFP assay. The data represent the mean ± SD. **e**, **f** Wild-type, H1.2 KO (1#), two ATM KO (1# and 3#) and two ATM/H1.2 double KO (5# and 6#) HeLa cells were analyzed by comet and colony formation assays. The tail moment of wild type cells at 12 h post treatment was normalized to 1. The data represent the mean ± SD. **g**, **h** Wild-type, H1.2 KO (1#), H1.2 KO (1#) with reintroduced wild type or S188A mutated H1.2, and ATM KO (1#) HeLa cells were analyzed by comet and colony formation assays. ATM KO (1#) HeLa cells with reintroduced wild-type or S188A mutated H1.2 were also analyzed by colony formation assay. The tail moment of wild-type cells at 10 min post treatment was normalized to 1. The data represent the mean ± SD. **i** A schematic model for the dynamic regulation of ATM by H1.2. In the absence of DNA damage, H1.2 binds to the chromatin and blocks the interactions between ATM and MRN to prevent the recruitment and activation of ATM. Upon DNA damage, PARP1 is activated to PARylate and displace H1.2 from chromatin, whereby ATM is permitted to be recruited and activated by MRN and DNA breaks. Activated ATM, which is amplified by an ATM-MDC1-MRN positive feedback loop, drives the DNA damage response through phosphorylation of a wide spectrum of substrates, including H2AX

To clarify the biological functions of H1.2 and its modifications, we investigated its role in DNA damage repair and cell survival. Global chromatin structure and cell survival were unaltered in H1.2 KO cells without DNA damage, as measured by micrococcal nuclease sensitivity and colony formation assays (Supplementary information, Figure S6a and b). Comet and colony formation assays further demonstrated that H1.2 KO cells exhibited increased repair activity and higher survival rates compared to wild-type, H1.3 or H1.4 KO cells (Fig. 7a, b; Supplementary information, Figure S6c and d). Interestingly, H1.3 and H1.4 KO cells showed moderately impaired cell survival compared to wild-type cells (Supplementary information, Figure S6d), suggesting that H1 variants may function differently in DNA repair. Similarly, stable H1.2 knockdown cells exhibited higher survival rates following etoposide treatment (Supplementary information, Figure S6e and f). In addition, stable over-expression of H1.2, but not H1.3 or H1.4, reduced the DNA repair efficiency (Supplementary information, Figure S6g). Over-expression of H1.2 also resulted in delayed removal of γ-H2AX, an indicator of DNA repair (Supplementary information, Figure S6h). We also used DR-GFP and pEJ5-GFP U2OS cells to measure the efficiencies of HR and NHEJ pathways, the two major pathways for repair of DSBs.^29^ As expected, H1.2 KO led to an increase in both the HR and NHEJ efficiencies (Fig. 7c, d; Supplementary information, Figure S6i and j). Consistently, over-expression of H1.2, rather than other H1 isoforms resulted in a significant decrease in repair activity in DR-GFP cells (Supplementary information, Figure S6k).

Finally, we demonstrate that H1.2 displacement and PARylation are indispensable for ATM-dependent DNA repair and cell survival. In accordance with the inhibitory role of H1.2 in the ATM-dependent DNA damage response and repair, ATM KO cells exhibited poor repair efficiencies and decreased cell survival, which could not be reversed by H1.2 KO (Fig. 7e, f; Supplementary information, Figure S7a–c). Consistently, resistance to etoposide treatment induced by H1.2 knockdown was restored by ATM or PARP1 inhibition, but not by DNA-PK inhibition (Supplementary information, Figure S7d–f). More importantly, rescue experiments showed that enhanced DNA repair efficiency and resistance to DNA damage treatment in H1.2 KO cells were suppressed by the reintroduction of mutated H1.2 (S188A), whereas wild-type H1.2 exhibited a moderate detrimental effect (Fig. 7g, h; Supplementary information, Figure S7g). In addition, reintroduction of either wild-type or mutated H1.2 could neither rescue nor aggravate impaired cell survival in ATM KO cells (Fig. 7h). Together, these results suggest that PARylation of H1.2 at S188 residue is indispensable for efficient DNA damage repair and cell survival.

## Discussion

Our data support a role for linker histone H1.2 in restraining ATM activity and protecting chromatin from aberrant ATM loading and activation. Upon DNA damage, H1.2 is PARylated and displaced from chromatin to permit full activation of ATM (Fig. 7i). We therefore propose a novel model whereby H1.2 functions as a molecular brake to ATM’s binding to MRN and that DNA damage-induced ATM activation requires both assembly of the MRN complex and release of H1.2.

Structural changes of chromatin may dictate ATM activation, but the facts that ATM can be activated when chromatin is either compacted (NaCl treatment) or decompacted (HDAC inhibitor treatment) suggest that additional factors are also involved in ATM activation. Various mechanisms have been reported to account for the regulation of ATM. For example, FoxO3a interacts with the FAT domain of ATM to regulate its dimerization and subsequent activation.^30^ The E3 ligases Chfr and RNF8 synergistically control histone ubiquitination and acetylation, leading to chromatin relaxation and ATM activation.^31^ A recent study found that DNA-PKcs directly phosphorylates ATM at multiple sites and inhibits ATM activity.^32^ In this study, we show ATM interacts with H1.2 via a specific fragment of its HEAT repeat domain, which critically regulates the binding of ATM substrates and regulators.^33–35^ This provides a structural basis for the direct inhibition of ATM activity by H1.2. Mechanistically, we demonstrate that H1.2, as a critical component of the chromatin, competes with MRN for the binding of ATM, which leads to impaired ATM interaction with MRN or its substrates. The existence of H1.2 as an inhibitory factor of ATM activation is supported by the theory that the DNA damage response should be tightly checked and spatiotemporally regulated to ensure optimized DNA repair.^36^ Most importantly, we showed that ATM activation correlates with H1.2 destabilization upon the application of non-DNA-damaging stimuli, such as HDAC inhibitors and NaCl. These results are supported by previous studies showing that the ATM-dependent DNA damage response can be activated without DNA breaks, for example, by persistent chromatin binding of DNA repair factors or local condensation of chromatin.^37,38^

It is now well appreciated that MRN activates ATM via multiple mechanisms and the interaction between ATM and the MRN complex is essential for ATM’s activation.^39^ The MRN complex is a bona fide sensor of DNA DSBs and amplifies ATM activation through a positive feedback loop.^40–45^ Here, we show that regulation of ATM by H1.2 is MRN-dependent. H1.2 associates with the MRN complex and inhibits MRN-dependent ATM activation without affecting the recruitment of MRN. This agrees with the earlier finding that localization of MRN to DNA damage sites is independent of ATM.^46^ These data also suggest that the previously reported ATM-dependent regulation of MRN recruitment may result in its relocalization to regions flanking DSBs. ATM can also be recruited and activated without DNA damage sensors,^47,48^ raising the possibility that H1.2 regulates the activation of ATM through other pathways. For example, it is recently reported that H1.2 inhibits H4K16 acetylation by promoting the expression of histone deacetylase SIRT1 and HDAC1.^49^ Since H4K16 acetylation has been demonstrated to be critical in regulating ATM activation,^50^ it is rational to speculate that H1.2 may also regulate ATM in this manner.

Chromatin structure and dynamics, for which linker histone H1 plays an essential role, are key factors in DNA repair and maintenance of genome integrity.^51,52^ However, how H1 participates in the DNA damage response and repair process remains elusive. We refined this concept and demonstrated the specific displacement and destabilization of linker histone H1.2 upon DNA damage. These findings further support the participation of both active recruitment and dissociation of signaling and repair factors in DNA damage-induced protein dynamics.^53^ Although H1.2 KO did not alter general chromatin structure or cell survival under unstressed conditions, our results, together with previous reports, show that deletion of H1.2 leads to cell cycle progression defects.^54^ This suggests that H1.2 may harbor critical functions in undamaged cells. Data derived from *Drosophila* models showed that H1 counters genome instability through inhibition of R-loop formation.^6^ This is in accord with our data that H1.2 potently inhibits ATM activation, because R-loops are known to activate ATM.^22^ H1 has been proposed to amplify DNA damage-induced ubiquitin signaling through RNF8-dependent ubiquitination and promote DNA repair, although a very recent study disputes the role of H1 and suggests that L3MBTL2 is required to amplify RNF8/RNF168-mediated ubiquitin signals.^9,55^ Hence it is possible that different H1 variants may function distinctly at different stages of the DNA damage response and repair. An example of this functional discrepancy is that H1.2 specifically regulates DSB-induced apoptosis, whereas H1.3 and H1.4 cannot.^11^ Together with these reports, our findings point to previously underestimated functions of H1 in DNA damage repair.

PARP1 is among the first proteins to respond to DNA damage, and its function in both SSB and DSB repair has been extensively characterized.^56^ Deletion or inhibition of PARP1 results in hypersensitivity to DNA damage inducers and compromised ATM activation.^19,57,58^ PARP1 modifies a set of proteins via the addition of ADP-ribose moieties to its substrates to alter protein–protein or protein–DNA interactions.^59^ In the present study, we found that DNA damage-induced H1.2 dynamics were regulated via PARylation of its C terminus. PARylation, unlike other post-translational modifications, is an immediate response to DNA damage, as PARP1 is activated within the first seconds of detecting damage.^53^ These data are in agreement with our proposal that H1.2 displacement precedes ATM activation, suggesting that H1 PARylation is a unique modification that regulates ATM activation and the DNA damage response. H1 is known to undergo various modifications upon DNA damage, including rapid dephosphorylation by the protein phosphatases PP1/PP2A, phosphorylation by DNA-PKcs and ubiquitination by RNF8/RNF168 following DNA damage treatment.^9,60,61^ Although we did not observe a functional relevance of these H1 post-translational modifications in ATM activation, PARylation of H1 may crosstalk with other modifications, which will be an interesting topic for future study.

Our present study has provided novel insight into the essential role of linker histone H1.2 destabilization in ATM activation and DNA damage repair. Further investigations into the chromatin perturbation that controls ATM activation are now warranted. Our identification of linker histone H1.2 as a novel PARP1 downstream target may help us better understand the functional mechanisms of ATM inhibition in cancer treatment and may lead to the discovery of new promising targets for cancer therapy.

## Materials and methods

### Cell culture, antibodies and reagents

HeLa, HCT116, HEK293T and U2OS cells were purchased from American Type Culture Collection (ATCC). NBS1 KO HeLa cells were kindly provided by Dr. Junjie Chen (MD Anderson Cancer Center, USA). Cells were cultured in DMEM or McCoy’s 5A medium supplemented with 10% (vol/vol) fetal bovine serum and 1% penicillin/streptomycin according to ATCC guidelines and maintained in a 37 °C incubator with a humidified, 5% CO_2_ atmosphere. The antibodies used in this study include: anti-ATM (GeneTex, GTX70103), anti-H1.2 (GeneTex, GTX122561), anti-GFP (MBL, M048-3), anti-HIS (MBL, PM032), anti-HA (MBL, M180-3), anti-FLAG (Sigma-Aldrich, F3165), anti-GST (APPLYGEN, C1303), anti-phospho-ATM (S1981; Cell Signaling, 5883), anti-phospho-NBS1 (S343; Cell Signaling, 3001), anti-phospho-SMC1 (S957; Cell Signaling, 58052), anti-SMC1 (Cell Signaling, 4802), anti-phospho-CHK2 (T68; Cell Signaling, 2197), anti-CHK2 (Cell Signaling, 3440), anti-phospho-p53 (S15; Cell Signaling, 9286), anti-phospho-H2AX (S139; Cell Signaling, 9718), anti-H2AX (Cell Signaling, 2595), anti-PARP1 (Cell Signaling, 9532), anti-GAPDH (Santa Cruz, sc-32233), anti-ATR (Santa Cruz, sc-1887), anti-DNA-PKcs (Santa Cruz, sc-1552), anti-NBS1 (Santa Cruz, sc-8580), anti-p53 (Santa Cruz, sc-126), anti-H1.4 (Santa Cruz, sc-34464), anti-cyclin E (Santa Cruz, sc-247), anti-H1.2 (for IP and ChIP; Abcam, ab17677), anti-H1.3 (Abcam, ab24174), anti-H3 (Abcam, ab1791), anti-H4 (Abcam, ab10158), anti-RAD50 (Abcam, ab89), anti-MRE11 (Abcam, ab12159), anti-phospho-H3 (S10; Abcam, ab5176), anti-ace-H3 (Active Motif, 39139), anti-H1 (Active Motif, 39707), anti-PAR (Trevigen, 4335-MC-100). Etoposide, adriamycin, cisplatin, hydroxyurea, oxaliplatin, thymidine, chloroquine (CHQ), Leptomycin B (LMB) and G418 were purchased from Sigma-Aldrich, and all other inhibitors, including Ku55933, Ku57788, PJ34, Oprozomib and MG132, were purchased from Selleckchem, USA.

### Plasmids

All plasmids were transfected with Lipofectamine 2000 (Life Technologies-Invitrogen, USA) according to the manufacturer’s instructions. H1.2, H1.3 and H1.4 cDNAs were amplified and cloned into the p3× FLAG-CMV-10 vector (Addgene, USA). Full-length H1.2 and various fragments (N-terminal domain, 1–35 aa; globular domain, 36–112 aa; C-terminal domain, 113–213 aa; ΔC1, 1–179 aa; ΔC2, 1–112+180–213 aa; ΔC, 1–112 aa) were cloned into the pEGFP-C2, pGEX-4T3 or pET28a vectors (Addgene, USA). The full-length FLAG-ATM expression construct was purchased from Addgene, USA. GST-ATM fragments (F1, 1–247 aa; F2, 250–522 aa; F3, 523–769 aa; F4, 722–1102 aa; F5, 1098–1371 aa; F6, 1245–1435 aa; F7, 1239–1770 aa; F8, 1764–2138 aa; F9, 2141–2428 aa; F10, 2427–2841 aa; F11, 2842–3056 aa; F12, 2682–3012 aa) were provided by Dr. Fabrizio d’Adda di Fagagna (FIRC Institute of Molecular Oncology Foundation, Italy). Site-specific mutations of H1.2 (T126/146/165A, T126/146/165E, E115A, S173A, S188A) were generated using a site-directed mutagenesis kit (Vazyme, China).

### CRISPR-Cas9 based gene-editing

H1 variant-specific KO HeLa cells were generated via Lipofectamine 2000 transfection of sgRNA constructs in a px459/Puro vector (Addgene, USA), as previously described.^62^ The sgRNA sequences targeted H1.2 (sequence 1: GGTACGCCTCGTAAGGCGTC, sequence 2: GGCTGGGGGTACGCCTCGTA), H1.3 (sequence 1: CGCAAGCGCTTTCTTAAGCG, sequence 2: GGTGTTTTTTCTGCGGGTGC), H1.4 (sequence 1: TTCACGGGAGTCTTCTCGGC, sequence 2: GCGGCCAAGCGCAAAGCGTC) and ATM (sequence 1: CTCTATCATGTTCTAGTTGA, sequence 2: TTGTTTCAGGATCTCGAATC, sequence 3: CGGCATTCAGATTCCAAACA).

### Chromatin fractionation

Cells were harvested into buffer I (50 mM HEPES pH 7.5, 150 mM NaCl and 1 mM EDTA) supplemented with 0.1% Triton X-100, 1% protease inhibitor cocktail (Roche Holding AG, Switzerland) and 2 μM PMSF and lysed on ice for 3 min. The supernatant was discarded and the pellet dissolved in buffer I supplemented with 200 μg/mL RNaseA and 1% protease inhibitor cocktail and incubated at room temperature for 30 min. The supernatant was discarded after centrifugation and the pellet was resuspended in buffer I, boiled in an equal volume of 2× SDS/PAGE sample buffer at 100 °C for 5 min and subjected to immunoblotting.

### Immunoprecipitation assay

Cells were harvested and lysed in NP-40 buffer (20 mM Tris·HCl pH 8.0, 137 mM NaCl, 1% NP-40, 10% glycerol, 2 mM EDTA, 1% protease inhibitor cocktail) for 30 min at 4 °C. After centrifugation, the supernatant was incubated with the indicated antibodies and protein G or A Sepharose slurry (GE Healthcare, USA) at 4 °C rotating overnight. For benzonase treatment, the supernatant was treated with benzonase (Millipore, USA) at 10 U/mL at 4 °C while rotating for 2 h before incubating with the antibodies. The beads were then washed and analyzed by immunoblotting.

### GST pull-down

GST or GST-tagged plasmids were transformed into *Escherichia coli* BL21 cells (TianGen, China) and induced with 0.1 mM IPTG (Sigma-Aldrich, USA) overnight at 28 °C and then purified using glutathione–Sepharose 4B beads (GE Healthcare, USA). HIS-tagged plasmids were transformed and induced in the same way, but purified using HIS agarose beads. Equal amounts of individual HIS-fusion protein were incubated with GST-fusion proteins (from *E. coli*) in TEN buffer (10 mM Tris·HCl pH 8.0, 1 mM EDTA, 100 mM NaCl) for 4 h at 4 °C. The samples were then washed three times in TEN buffer by centrifugation at 94 × *g* at 4 °C for 1 min and the precipitated components were analyzed by immunoblotting.

### In vitro phosphorylation assay

Full-length ATM and ATR plasmids were transfected into HEK293T cells and the ATM or ATR proteins were immunoprecipitated using FLAG-conjugated M2 agarose beads 48 h after transfection. Beads were first washed with lysis buffer (without NP-40) and then twice in kinase buffer (10 mM Tris·HCl pH 7.4, 150 mM NaCl, 10 mM MgCl_2_, 0.5 mM DTT) before elution with the FLAG peptide. The eluents were added to kinase buffer and incubated with different substrates and purified proteins. The reaction was initiated by adding 30 μM ATP (final concentration), and incubated at 30 °C for 1 h. The samples were subjected to CBB staining or immunoblotting after adding 5× SDS/PAGE sample buffer and boiling at 100 °C for 5 min.

### In vitro PARylation assay

HIS-H1.2 was subjected to in vitro PARylation at room temperature for 30 min or the indicated time in a reaction buffer (50 mM Tris·HCl pH 8, 25 mM MgCl_2_, 50 mM NaCl) supplemented with 200 μM NAD^+^, activated DNA and PARP1 enzyme (Thermo Fisher, or immunoprecipitated from HET293T cells). The reaction was stopped by adding 5× SDS/PAGE sample buffer and the samples were analyzed by immunoblotting.

### Laser micro-irradiation-coupled live-cell imaging

Laser micro-irradiation was performed as previously descried.^63^ Briefly, cells were grown on a glass-bottomed dish and locally irradiated with a 365 nm pulsed nitrogen UV laser (16 Hz pulse, 41% laser output) generated from a micropoint system (Andor). This system was directly coupled to the epifluorescence path of the Nikon A1 confocal imaging system and time-lapse images were captured every 10 s for the indicated time. Signal intensity of the irradiation path from more than 50 cells was calculated using an ImageJ software (version 1.51j8).

### Immunofluorescence

Cells were fixed with 4% paraformaldehyde and permeabilized with 0.1% Triton X-100, followed by blocking with 1% BSA. For in situ detergent extraction, cells were lysed in the dish in buffer I (50 mM HEPES pH 7.5, 150 mM NaCl and 1 mM EDTA) supplemented with 0.1% Triton X-100 for 10 min on ice before fixation. The cells were then incubated with the indicated primary antibodies at 4 °C overnight. After being washed three times with 1% BSA, the slides were exposed to a FITC/TRITC-conjugated secondary antibody for 2 h at room temperature in the dark and then washed three times with blocking buffer. The samples were then embedded in DAPI and observed under an Olympus FV1000-IX81 confocal microscope. About 200 cells were analyzed for quantification and the experiments were repeated independently for at least three times.

### Comet assay

Comet assays were performed as previously described.^64^ Briefly, cells were treated with 40 μM etoposide for 2 h and released for the indicated time. The collected cells were then mixed gently with pre-melted low-temperature-melting agarose at a volume ratio of 1:1 (v/v) and spread on glass slides. The slides were then submerged in pre-cooled lysis buffer at 4 °C for 90 min. After rinsing, the slides were subjected to electrophoresis at 1.0 V/cm for 20 min, and then stained with propidium iodide (PI). Fluorescent images of ≥ 100 nuclei were captured under an Olympus FV1000-IX81 Confocal Microscope (Tokyo, Japan). The images were analyzed for tail moment using CASP (Comet Assay Software Project) version 1.2.2. Quantification of tail moment was calculated by measuring the tail length and amount of DNA (quantified by PI intensity) in the tail.

### DR-GFP assay and EJ5-GFP assay

DR-GFP or pEJ5-GFP U2OS cells with a single copy of DR-GFP in a random locus were transfected with HA-I-*Sce*I and other indicated plasmids 24 h before HA-I-*Sce*I transfection if necessary. Cells were harvested 48 h after HA-I-*Sce*I transfection and subjected to flow cytometric analysis. The percentage of GFP-positive cells, which indicated HR-mediated or NHEJ-mediated DSB repair efficiency, was determined. The mean values were obtained from three independent experiments.

### Colony formation assay

Cells were seeded in six-well plates and after 24 h, were exposed to etoposide at the indicated concentrations for 2 h (in some cases inhibitors were added 1 h prior to etoposide treatment). The cells were then washed three times with serum-free medium and then re-cultured in fresh medium. After approximately 10 days culture under normal conditions, cell colonies were visualized by crystal violet staining and colonies consisting of > 50 cells were counted.

### Statistics

Data were analyzed by Student’s *t* test. A *p* < 0.05 was considered statistically significant (N.S., *p* > 0.05, *p < 0.05, **p *<* 0.01, ***p < 0.001). Three or more independent experiments were performed in all cases. The data represent the mean ± SD. Other materials and methods are described in Supplementary information, Data S1.

## Electronic supplementary material

Supplementary information, Figure S1

Supplementary information, Figure S2

Supplementary information, Figure S3

Supplementary information, Figure S4

Supplementary information, Figure S5

Supplementary information, Figure S6

Supplementary information, Figure S7

Supplementary information, Data S1
