# Supplementary material for: Destabilization of linker histone H1.2 is essential for ATM activation and DNA damage repair
Source: Cell Res. 2018 May 29;28(7):756–70. doi: 10.1038/s41422-018-0048-0 (PMC6028381; doi:10.1038/s41422-018-0048-0)
Supplement: Supplementary file 1 — Supplementary information, Figure S1 [file 41422_2018_48_MOESM1_ESM.pdf]

Figure S1

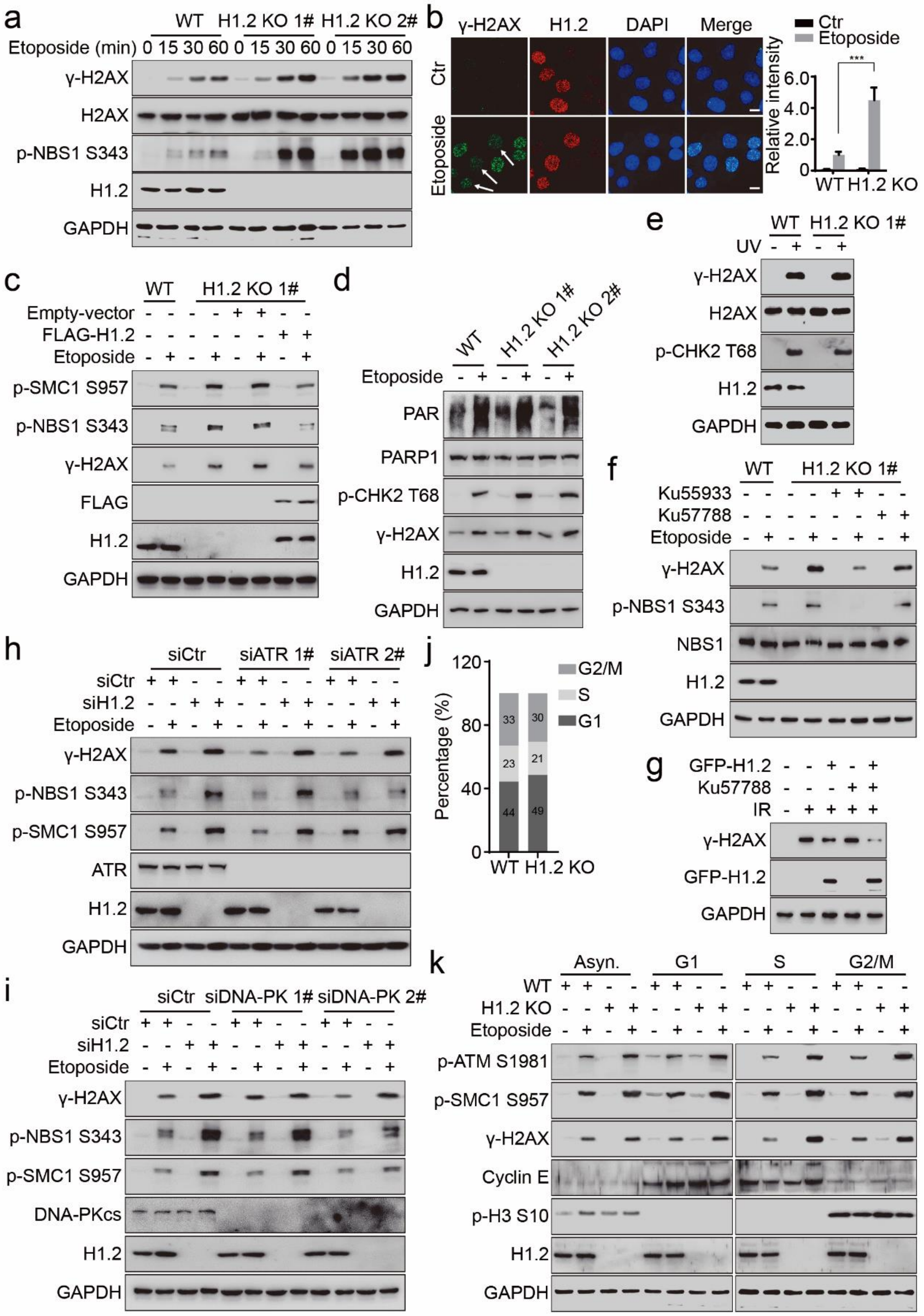
a
WT
H1.2 KO 1#
H1.2 KO 2#
Etoposide (min) 0 15 30 60 0 15 30 60 0 15 30 60
γ-H2AX
H2AX
p-NBS1 S343
H1.2
GAPDH
b
γ-H2AX
H1.2
DAPI
Merge
Ctr
Etoposide
Ctr
Etoposide
Relative intensity
6.0
4.0
2.0
0
***
WT
H1.2 KO
c
WT
H1.2 KO 1#
Empty-vector
FLAG-H1.2
Etoposide
p-SMC1 S957
p-NBS1 S343
γ-H2AX
FLAG
H1.2
GAPDH
d
WT
H1.2 KO 1#
H1.2 KO 2#
Etoposide
PAR
PARP1
p-CHK2 T68
γ-H2AX
H1.2
GAPDH
e
WT
H1.2 KO 1#
UV
γ-H2AX
H2AX
p-CHK2 T68
H1.2
GAPDH
f
WT
H1.2 KO 1#
Ku55933
Ku57788
Etoposide
γ-H2AX
p-NBS1 S343
NBS1
H1.2
GAPDH
g
GFP-H1.2
Ku57788
IR
γ-H2AX
GFP-H1.2
GAPDH
h
siCtr
siATR 1#
siATR 2#
siCtr
siH1.2
Etoposide
γ-H2AX
p-NBS1 S343
p-SMC1 S957
ATR
H1.2
GAPDH
j
120
80
40
0
Percentage (%)
G2/M
S
G1
33
23
44
30
21
49
WT
H1.2 KO
i
siCtr
siDNA-PK 1#
siDNA-PK 2#
siCtr
siH1.2
Etoposide
γ-H2AX
p-NBS1 S343
p-SMC1 S957
DNA-PKcs
H1.2
GAPDH
k
Asyn.
G1
S
G2/M
WT
H1.2 KO
Etoposide
p-ATM S1981
p-SMC1 S957
γ-H2AX
Cyclin E
p-H3 S10
H1.2
GAPDH

Figure S1. Linker histone H1.2 inhibits ATM-dependent DNA damage response

a WT and H1.2 KO (1# and 2#) HeLa cells were treated with 40 μM etoposide for the indicated times and analyzed by immunoblotting. **b** WT and H1.2 KO (1#) HeLa cells were mixed and treated with 40 μM etoposide for 2 h before analysis by immunofluorescence assay. The arrows indicate representative cells. The γ-H2AX intensity in the etoposide treated WT cells was normalized to 1. The data represent the mean ± SD. Scale bars, 10 μm. **c** WT and H1.2 KO (1#) HeLa cells were transfected with the indicated plasmids with or without 40 μM etoposide treatment for 1 h and analyzed by immunoblotting. **d** WT and H1.2 KO (1# and 2#) HeLa cell clones were treated with 40 μM etoposide for 15 min and analyzed by immunoblotting. **e** WT or H1.2 KO (1#) HeLa cells were exposed to 100 mJ ultraviolet light and were collected 1 h after exposure and analyzed by immunoblotting. **f** WT or H1.2 KO (1#) HeLa cells were treated with 40 μM etoposide for 1 h. H1.2 KO HeLa cells were treated with 20 μM Ku55933 or 1 μM Ku57788 for 1 h prior to etoposide treatment and analyzed by immunoblotting. **g** HeLa cells were transfected with GFP-H1.2 and exposed to 10 Gy IR with or without 2 h prior exposure to 2 μM Ku57788. Cells were collected 1 h post IR and analyzed by immunoblotting. **h**, **i** HeLa cells were transfected with the indicated siRNAs and treated with 40 μM etoposide for 1 h and then analyzed by immunoblotting. **j** WT or H1.2 KO (1#) HeLa cells were subjected to flow cytometry analysis. **k** WT or H1.2 KO (1#) HeLa cells were synchronized into different phases of cell cycle and treated with 40 μM etoposide for 1 h and analyzed by immunoblotting. Asyn. indicates cells that are asynchronized. G1, S and G2/M indicate cells that are synchronized into these cell cycle phases.
