# Supplementary material for: Destabilization of linker histone H1.2 is essential for ATM activation and DNA damage repair
Source: Cell Res. 2018 May 29;28(7):756–70. doi: 10.1038/s41422-018-0048-0 (PMC6028381; doi:10.1038/s41422-018-0048-0)
Supplement: Supplementary file 2 — Supplementary information, Figure S2 [file 41422_2018_48_MOESM2_ESM.pdf]

Figure S2

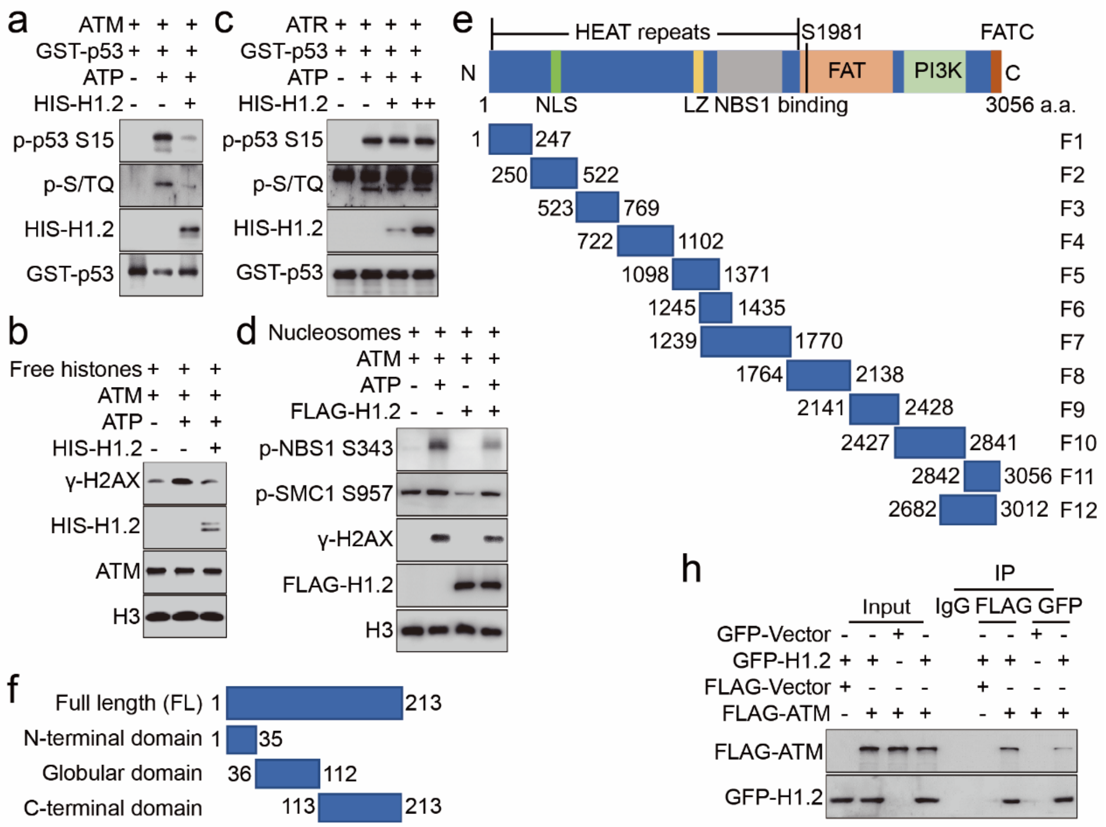

g

Names	Score	# Unique peptides	Sequence coverage %
PRKDC	1703.6901	50	12.43
XRCC6	1646.14303	25	45.32
PARP1	1643.87002	38	34.22
XRCC5	1095.16973	22	33.88
ELAVL1	618.245473	10	37.12
TOP1	602.672467	17	21.57
PRMT1	602.627515	12	36.01
**HIST1H1C**	458.321891	2	28.17
CARM1	264.704518	7	12.17
CHD4	83.459234	1	1.52

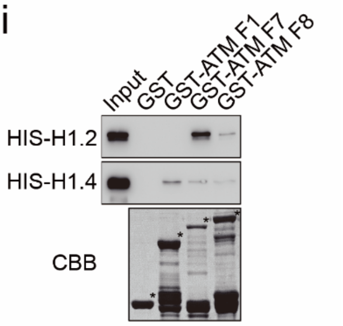

Figure S2. Linker Histone H1.2 interacts with ATM and directly inhibits its activity

a, **c** An N-terminal GST-p53 (1-99 aa) peptide was used as substrate for *in vitro* phosphorylation assay with or without HIS-H1.2. **b** Free histones extracted from HeLa cells were used as substrates for *in vitro* phosphorylation assay with or without recombinant HIS-H1.2. **d** HeLa cells were transfected with FLAG-H1.2 or an empty vector and mononucleosomes were extracted and subjected to *in vitro* phosphorylation assay. **e** Organization of the ATM domains and the generated fragments. **f** Fragments of H1.2. **g** Summary of the proteins identified by mass spectrometry detailed in Figure 2F. Gene names, protein score, numbers of unique peptides and sequence coverage are shown. Name in bold indicates the desired protein. **h** HEK293T cells were transfected with the indicated plasmids and subjected to Co-IP assay with the indicated antibodies. **i** GST alone or GST ATM fragments were incubated with HIS-H1.2 or HIS-H1.4 for GST pull-down assay. * indicates specific protein bands.
