# Supplementary material for: Destabilization of linker histone H1.2 is essential for ATM activation and DNA damage repair
Source: Cell Res. 2018 May 29;28(7):756–70. doi: 10.1038/s41422-018-0048-0 (PMC6028381; doi:10.1038/s41422-018-0048-0)
Supplement: Supplementary file 3 — Supplementary information, Figure S3 [file 41422_2018_48_MOESM3_ESM.pdf]

Figure S3

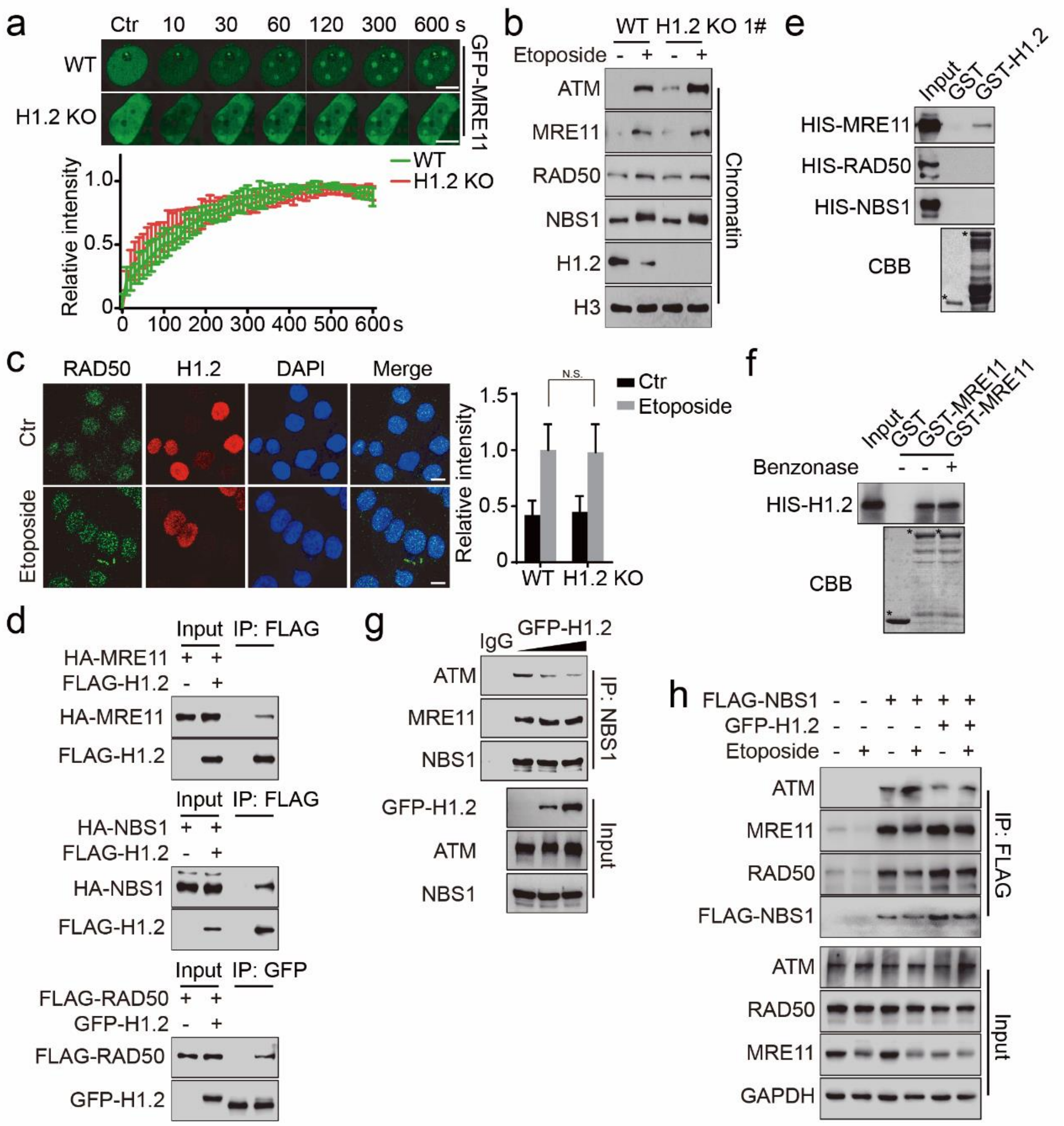

a
Ctr 10 30 60 120 300 600 s
WT
H1.2 KO
GFP-MRE11
WT
H1.2 KO
Relative intensity
1.0
0.5
0
0 100 200 300 400 500 600 s
b
WT H1.2 KO 1#
Etoposide - + - +
ATM
MRE11
RAD50
NBS1
H1.2
H3
Chromatin
e
Input GST GST-H1.2
HIS-MRE11
HIS-RAD50
HIS-NBS1
CBB
c
RAD50 H1.2 DAPI Merge
Ctr
Etoposide
N.S.
Ctr
Etoposide
Relative intensity
1.5
1.0
0.5
0
WT H1.2 KO
f
Input GST GST-MRE11 GST-MRE11
Benzonase - - +
HIS-H1.2
CBB
d
Input IP: FLAG
HA-MRE11 + +
FLAG-H1.2 - +
HA-MRE11
FLAG-H1.2
Input IP: FLAG
HA-NBS1 + +
FLAG-H1.2 - +
HA-NBS1
FLAG-H1.2
Input IP: GFP
FLAG-RAD50 + +
GFP-H1.2 - +
FLAG-RAD50
GFP-H1.2
g
IgG GFP-H1.2
ATM
MRE11
NBS1
IP: NBS1
GFP-H1.2
ATM
NBS1
Input
h
FLAG-NBS1 - - + + + +
GFP-H1.2 - - - - + +
Etoposide - + - + - +
ATM
MRE11
RAD50
FLAG-NBS1
IP: FLAG
ATM
RAD50
MRE11
GAPDH
Input

Figure S3. Linker histone H1.2 inhibits ATM recruitment and activation by interacting with MRN

a WT and H1.2 KO (1#) HeLa cells were transfected with GFP-MRE11 and subjected to laser micro-irradiation-coupled live-cell imaging. Images were taken very 10 s for 10 min and the relative intensity of the irradiation path signal was shown. The data represent the mean ± SD. Scale bars, 10 μm. **b** WT and H1.2 KO (1#) HeLa cells were treated with 40 μM etoposide for 1 h. Chromatin was fractionated and analyzed by immunoblotting. **c** WT and H1.2 KO (1#) HeLa cells were mixed and then treated with 40 μM etoposide for 2 h and subjected to immunofluorescence assay with the indicated antibodies or labeled with DAPI. Quantifications of signal intensity are shown. The data represent the mean ± SD. Scale bars, 10 μm. **d** HEK293T cells were transfected with the indicated plasmids and analyzed by co-IP followed by immunoblotting. **e** GST alone or GST-H1.2 was incubated with HIS-MRE11, RAD50 and NBS1 for GST pull-down assay. * indicates specific protein bands. **f** GST alone or GST-MRE11 was incubated with HIS-H1.2 for GST pull-down assay with or without benzonase treatment. * indicates specific protein bands. **g** HeLa cells were transfected with an increasing amount of GFP-H1.2, and the whole cell lysates were immunoprecipitated with NBS1 or IgG antibody and subjected to immunoblotting. **h** HeLa cells were transfected with the indicated plasmids and treated with 40 μM etoposide for 1 h. Chromatin extracts were prepared and analyzed by co-IP followed by immunoblotting.
