# Supplementary material for: Destabilization of linker histone H1.2 is essential for ATM activation and DNA damage repair
Source: Cell Res. 2018 May 29;28(7):756–70. doi: 10.1038/s41422-018-0048-0 (PMC6028381; doi:10.1038/s41422-018-0048-0)
Supplement: Supplementary file 4 — Supplementary information, Figure S4 [file 41422_2018_48_MOESM4_ESM.pdf]

Figure S4

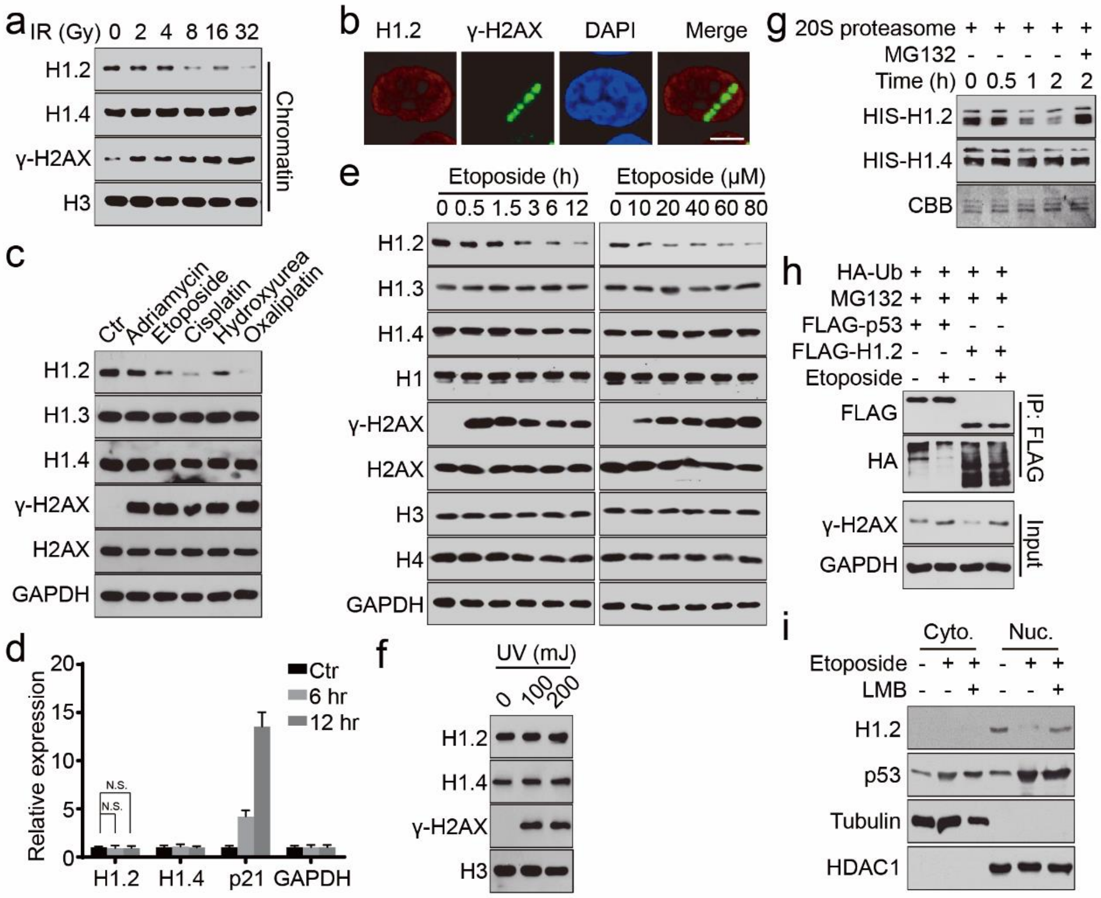
a
IR (Gy) 0 2 4 8 16 32
H1.2
H1.4
γ-H2AX
H3
Chromatin
b
H1.2
γ-H2AX
DAPI
Merge
c
Ctr
Adriamycin
Etoposide
Cisplatin
Hydroxyurea
Oxaliplatin
H1.2
H1.3
H1.4
γ-H2AX
H2AX
GAPDH
d
Relative expression
20
15
10
5
0
N.S.
N.S.
Ctr
6 hr
12 hr
H1.2
H1.4
p21
GAPDH
e
Etoposide (h)
0 0.5 1.5 3 6 12
Etoposide (μM)
0 10 20 40 60 80
H1.2
H1.3
H1.4
H1
γ-H2AX
H2AX
H3
H4
GAPDH
f
UV (mJ)
0 100 200
H1.2
H1.4
γ-H2AX
H3
g
20S proteasome + + + + +
MG132 - - - - +
Time (h) 0 0.5 1 2 2
HIS-H1.2
HIS-H1.4
CBB
h
HA-Ub + + + +
MG132 + + + +
FLAG-p53 + + - -
FLAG-H1.2 - - + +
Etoposide - + - +
FLAG
HA
IP: FLAG
γ-H2AX
GAPDH
Input
i
Cyto.
Nuc.
Etoposide - + + - + +
LMB - - + - - +
H1.2
p53
Tubulin
HDAC1

Figure S4. Linker histone H1.2 is rapidly displaced and degraded upon DNA damage

a HeLa cells were exposed to different doses of irradiation (IR) before collection after 30 min and analyzed by immunoblotting. **b** HeLa cells were subjected to laser micro-irradiation and fixed immediately before immunofluorescence assay. Cells were stained with the indicated antibodies or DAPI. Scale bars, 10 μm. **c** HCT116 cells were treated with 1 μM adriamycin, 20 μM etoposide, 10 μM cisplatin, 2 mM hydroxyurea or 10 μM oxaliplatin for 12 h and analyzed by immunoblotting. **d** HCT116 cells were treated with 20 μM etoposide for the indicated time and total RNA was extracted and H1.2, H1.4, p21 and GAPDH relative gene expression was analyzed by real-time PCR. The data represent the mean ± SD. **e** HeLa cells were treated with 20 μM etoposide for the indicated time or to increasing concentrations of etoposide for 12 h and analyzed by immunoblotting. **f** HeLa cells were exposed to different doses of ultraviolet (UV) radiation. Cells were collected 1 h after exposure and analyzed by immunoblotting. **g** Recombinant HIS-H1.2 or H1.4 was incubated with 20S proteasome substrate for the indicated time, with or without 10 μM MG132 for *in vitro* degradation assay. **h** HCT116 cells were transfected with the indicated plasmids and treated with 20 μM etoposide for 12 h. Cells were extracted after treatment of MG132 at 1 μM for 4 h and subjected to immunoprecipitation using FLAG-conjugated M2 beads. **i** HeLa cells were treated with etoposide at 20 μM for 12 h with or without leptomycin B (LMB) at 1 μM. Cytoplasmic (Cyto.) and nucleic (Nuc.) proteins were fractioned and subjected to immunoblotting.
