# Supplementary material for: Destabilization of linker histone H1.2 is essential for ATM activation and DNA damage repair
Source: Cell Res. 2018 May 29;28(7):756–70. doi: 10.1038/s41422-018-0048-0 (PMC6028381; doi:10.1038/s41422-018-0048-0)
Supplement: Supplementary file 5 — Supplementary information, Figure S5 [file 41422_2018_48_MOESM5_ESM.pdf]

Figure S5

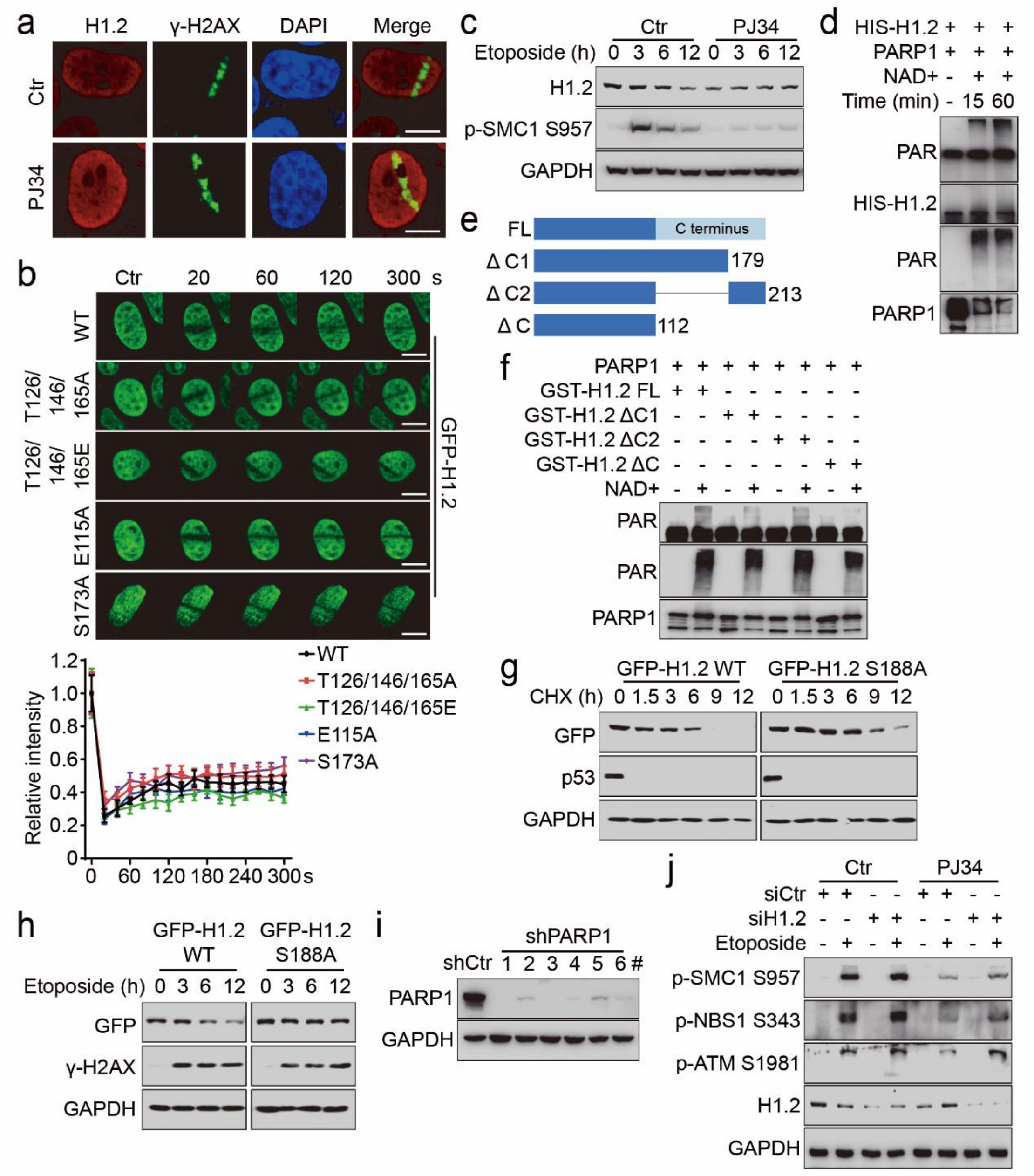

a
H1.2
γ-H2AX
DAPI
Merge
Ctr
PJ34
b
Ctr
20
60
120
300 s
WT
T126/146/165A
T126/146/165E
E115A
S173A
GFP-H1.2
Relative intensity
WT
T126/146/165A
T126/146/165E
E115A
S173A
0 60 120 180 240 300 s
c
Ctr
PJ34
Etoposide (h) 0 3 6 12 0 3 6 12
H1.2
p-SMC1 S957
GAPDH
d
HIS-H1.2 + + +
PARP1 + + +
NAD+ - + +
Time (min) - 15 60
PAR
HIS-H1.2
PAR
PARP1
e
FL
C terminus
Δ C1
179
Δ C2
213
Δ C
112
f
PARP1 + + + + + + + +
GST-H1.2 FL + + - - - - - -
GST-H1.2 ΔC1 - - + + - - - -
GST-H1.2 ΔC2 - - - - + + - -
GST-H1.2 ΔC - - - - - - + +
NAD+ - + - + - + - +
PAR
PAR
PARP1
g
GFP-H1.2 WT
GFP-H1.2 S188A
CHX (h) 0 1.5 3 6 9 12 0 1.5 3 6 9 12
GFP
p53
GAPDH
h
GFP-H1.2 WT
GFP-H1.2 S188A
Etoposide (h) 0 3 6 12 0 3 6 12
GFP
γ-H2AX
GAPDH
i
shCtr
shPARP1
1 2 3 4 5 6 #
PARP1
GAPDH
j
Ctr
PJ34
siCtr + + - - + + - -
siH1.2 - - + + - - + +
Etoposide - + - + - + - +
p-SMC1 S957
p-NBS1 S343
p-ATM S1981
H1.2
GAPDH

Figure S5. PARylation of linker histone H1.2 is required for its displacement and ATM activation

a HeLa cells were treated with or without 5 μM PJ34 for 1 h, subjected to laser micro-irradiation and fixed immediately before immunofluorescence assay. Cells were stained with the indicated antibodies or DAPI. Scale bars, 10 μm. **b** HeLa cells were transfected with the indicated WT or mutated GFP-H1.2 plasmids and subjected to laser micro-irradiation. Images were captured every 20 s for 5 min and quantifications of the IR path signal intensity are shown. The data represent the mean ± SD. Scale bars, 10 μm. **c** HeLa cells were treated with 20 μM etoposide for the indicate times, with or without 5 μM PJ34 for 1 h prior to etoposide treatment and then analyzed by immunoblotting. **d** Recombinant HIS-H1.2 was incubated with or without NAD^+ and PARP1 as indicated. The samples were analyzed by immunoblotting after *in vitro* PARylation assay. **e** Location of the H1.2 C-terminal deletion mutants. **f** Recombinant full-length (FL), C1-deleted (ΔC1), C2-deleted (ΔC2) or C-deleted (ΔC) GST-H1.2 were assessed for *in vitro* PARylation assay. **g**, **h** HeLa cells were transfected with the indicated plasmids and treated with 1 μM CHX or 20 μM etoposide for the indicated times before analyzed by immunoblotting. **i** HeLa cells were stably transfected with shRNAs targeting PARP1 and several clones (1-6#) were selected for immunoblotting to determine the efficiency of PARP1 knockdown. **j** HeLa cells were transfected with the indicated siRNAs and treated with 40 μM etoposide for 15 min with or without 5 μM PJ34 for 1 h and then analyzed by immunoblotting.
