# Supplementary material for: Destabilization of linker histone H1.2 is essential for ATM activation and DNA damage repair
Source: Cell Res. 2018 May 29;28(7):756–70. doi: 10.1038/s41422-018-0048-0 (PMC6028381; doi:10.1038/s41422-018-0048-0)
Supplement: Supplementary file 6 — Supplementary information, Figure S6 [file 41422_2018_48_MOESM6_ESM.pdf]

Figure S6

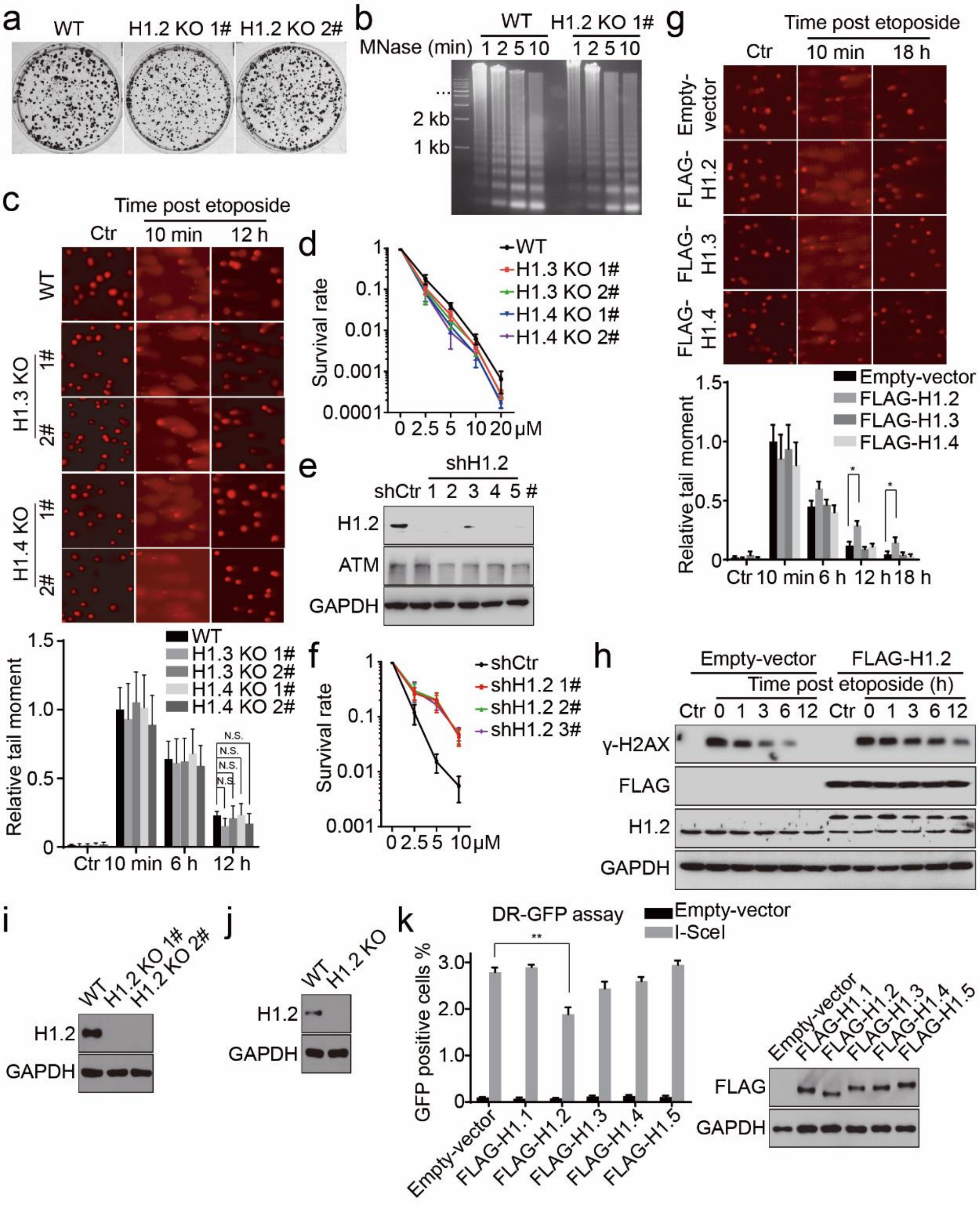
a
WT
H1.2 KO 1#
H1.2 KO 2#
b
WT
H1.2 KO 1#
MNase (min)
1 2 5 10
1 2 5 10
2 kb
1 kb
c
Time post etoposide
Ctr
10 min
12 h
WT
H1.3 KO
1#
2#
H1.4 KO
1#
2#
Relative tail moment
WT
H1.3 KO 1#
H1.3 KO 2#
H1.4 KO 1#
H1.4 KO 2#
N.S.
Ctr 10 min 6 h 12 h
d
Survival rate
WT
H1.3 KO 1#
H1.3 KO 2#
H1.4 KO 1#
H1.4 KO 2#
0 2.5 5 10 20 μM
e
shH1.2
shCtr 1 2 3 4 5 #
H1.2
ATM
GAPDH
f
Survival rate
shCtr
shH1.2 1#
shH1.2 2#
shH1.2 3#
0 2.5 5 10μM
g
Time post etoposide
Ctr
10 min
18 h
Empty-vector
FLAG-H1.2
FLAG-H1.3
FLAG-H1.4
Relative tail moment
Empty-vector
FLAG-H1.2
FLAG-H1.3
FLAG-H1.4
Ctr 10 min 6 h 12 h 18 h
h
Empty-vector
FLAG-H1.2
Time post etoposide (h)
Ctr 0 1 3 6 12
Ctr 0 1 3 6 12
γ-H2AX
FLAG
H1.2
GAPDH
i
WT
H1.2 KO 1#
H1.2 KO 2#
H1.2
GAPDH
j
WT
H1.2 KO
H1.2
GAPDH
k
DR-GFP assay
Empty-vector
I-SceI
GFP positive cells %
Empty-vector
FLAG-H1.1
FLAG-H1.2
FLAG-H1.3
FLAG-H1.4
FLAG-H1.5
FLAG
GAPDH

Figure S6. Linker histone H1.2 inhibits DNA damage repair and cell survival

a WT and H1.2 KO (1# and 2#) HeLa cell were seeded into 6-well plates (500 cells per well) and subjected to colony formation assay. **b** WT and H1.2 KO (1#) HeLa cells were subjected to micrococcal nuclease (MNase) sensitivity assay. **c** WT, H1.3 KO (1# and 2#) and H1.4 KO (1# and 2#) HeLa cells were subjected to comet assay. The cells were collected at the indicated times after treatment with 40 μM etoposide for 2 h. The tail moment of WT cells at 10 min post treatment was normalized to 1 and the quantification results are shown. The data represent the mean ± SD. **d** WT, H1.3 KO (1# and 2#) and H1.4 KO (1# and 2#) HeLa cells were subjected to colony formation assay after treatment with different doses of etoposide for 2 h. The data represent the mean ± SD. **e** HeLa cells were stably transfected with shRNAs targeting H1.2 and several clones (1-5#) were selected for immunoblotting to determine the knockdown efficiency. **f** H1.2 stable-knockdown (1-3#) or non-specific knockdown HeLa cells were treated with the indicated dose of etoposide for 2 h and subjected to colony formation assay. **g** HeLa cells with stably over-expressed FLAG-H1.2, H1.3, H1.4 or an empty vector were subject to comet assay. The tail moment of the empty vector cells at 10 min post treatment was normalized to 1. The data represent the mean ± SD. **h** HeLa cells were transfected with FLAG-H1.2 or an empty vector and were separately treated with 40 μM etoposide for 1 h and released at the indicated times. **i**, **j** Immunoblots for H1.2 and GAPDH in WT or H1.2 KO DR-GFP and pEJ5-GFP cells. **k** DR-GFP U2OS cells were transfected with the indicated plasmids and subjected to DR-GFP assay. The data represent the mean ± SD.
