# Supplementary material for: Destabilization of linker histone H1.2 is essential for ATM activation and DNA damage repair
Source: Cell Res. 2018 May 29;28(7):756–70. doi: 10.1038/s41422-018-0048-0 (PMC6028381; doi:10.1038/s41422-018-0048-0)
Supplement: Supplementary file 7 — Supplementary information, Figure S7 [file 41422_2018_48_MOESM7_ESM.pdf]

Figure S7

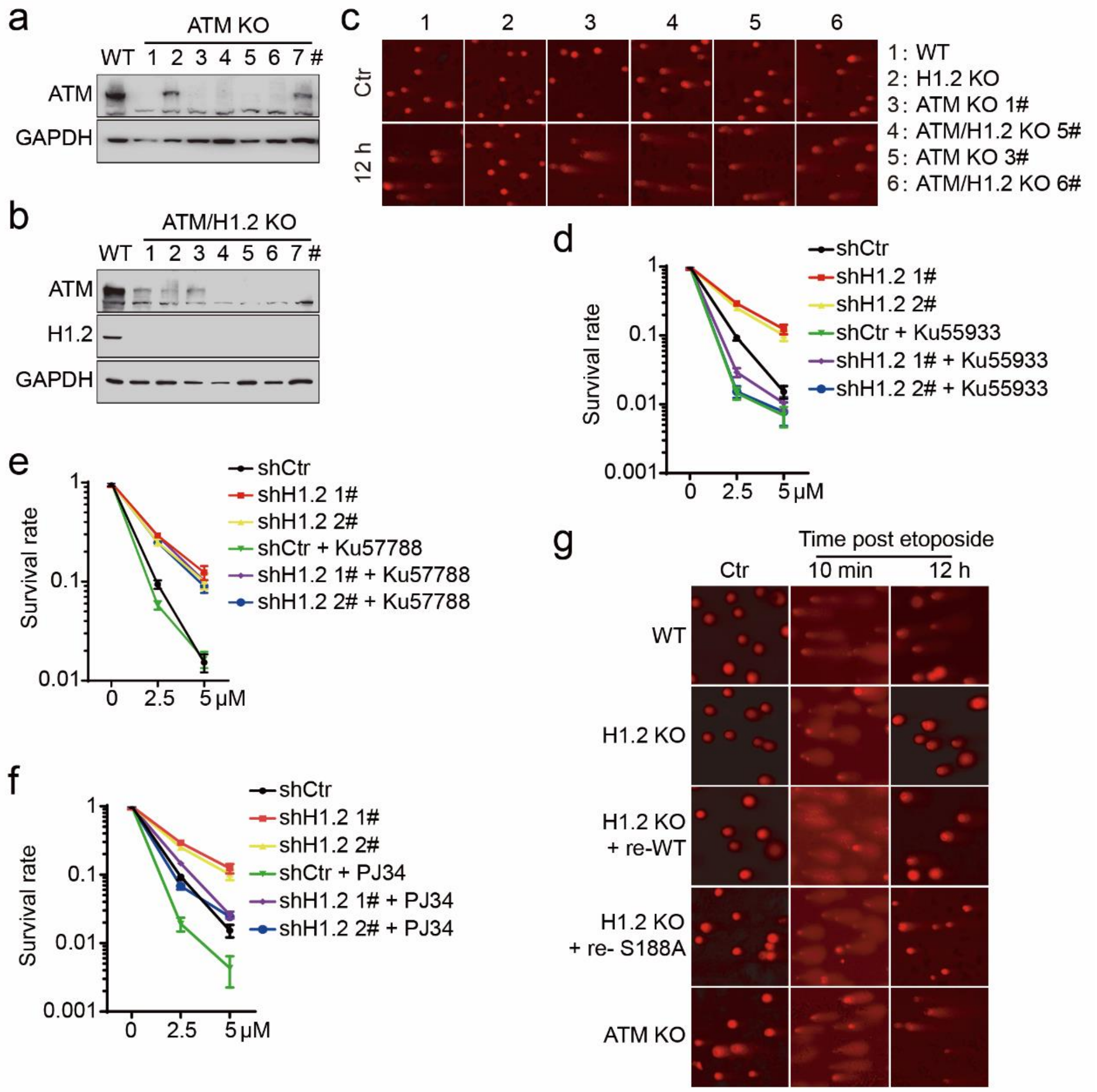
a
ATM KO
WT 1 2 3 4 5 6 7 #
ATM
GAPDH
b
ATM/H1.2 KO
WT 1 2 3 4 5 6 7 #
ATM
H1.2
GAPDH
c
1 2 3 4 5 6
Ctr
12 h
1: WT
2: H1.2 KO
3: ATM KO 1#
4: ATM/H1.2 KO 5#
5: ATM KO 3#
6: ATM/H1.2 KO 6#
d
Survival rate
1
0.1
0.01
0.001
0 2.5 5 μM
shCtr
shH1.2 1#
shH1.2 2#
shCtr + Ku55933
shH1.2 1# + Ku55933
shH1.2 2# + Ku55933
e
Survival rate
1
0.1
0.01
0 2.5 5 μM
shCtr
shH1.2 1#
shH1.2 2#
shCtr + Ku57788
shH1.2 1# + Ku57788
shH1.2 2# + Ku57788
f
Survival rate
1
0.1
0.01
0.001
0 2.5 5 μM
shCtr
shH1.2 1#
shH1.2 2#
shCtr + PJ34
shH1.2 1# + PJ34
shH1.2 2# + PJ34
g
Time post etoposide
Ctr 10 min 12 h
WT
H1.2 KO
H1.2 KO + re-WT
H1.2 KO + re- S188A
ATM KO

Figure S7. Linker histone H1.2 dissociation and destabilization is indispensable for DNA repair and cell survival

a Verification of ATM KO (KO) via CRISPR-Cas9 targeting in WT HeLa cells by immunoblotting, and efficiencies of clone 2# and 7# were poor. **b** Verification of ATM KO in H1.2 KO (1#) HeLa cells using CRISPR-Cas9 targeting by immunoblotting, and efficiencies of clone 1-3# were poor. **c** Representative images of the comet assay detailed in Figure 7**e**. **d-f** H1.2 stable-knockdown (1# and 2#) or control knockdown HeLa cells were treated with different doses of etoposide for 2 h with or without prior treatment with 20 μM Ku55933, 2 μM Ku57788 or 5 μM PJ34 for 1 h and then subjected to colony formation assay. The data represent the mean ± SD. **g** Representative images of the comet assay detailed in Figure 7**g**.
