# Supplementary material for: Destabilization of linker histone H1.2 is essential for ATM activation and DNA damage repair
Source: Cell Res. 2018 May 29;28(7):756–70. doi: 10.1038/s41422-018-0048-0 (PMC6028381; doi:10.1038/s41422-018-0048-0)
Supplement: Supplementary file 8 — Supplementary information, Data S1 [file 41422_2018_48_MOESM8_ESM.pdf]

Supplementary information, Data S1

Materials and Methods

siRNA and shRNA

The following siRNAs were used to silence target genes by transfecting with Lipofectamine 2000 or 3000 (Life Technologies-Invitrogen, USA) according to the manufacturer's instructions:

ATM siRNA 1: UGAAGUCCAUUGCUAAUCATT

ATM siRNA 2: GCGCCUGAUUCGAGAUCCUTT

DNA-PKcs siRNA 1: GGGCGCUAAUCGUACUGAATT

DNA-PKcs siRNA 2: GAUCGCACCUUACUCUGUUTT

ATR siRNA 1: GCCAAGACAAAUUCUGUGUTT

ATR siRNA 2: CCUCCGUGAUGUUGCUUGATT

PARP1 siRNA 1: GAAAGUGUGUUCAACUAAUUU

PARP1 siRNA 2: AAGAUAGAGCGUGAAGGCGAA

MRE11 siRNA: GGAGGUACGUCGUUUCAGA

H1.2 siRNA: AAGAGCGUAGCGGAGUUUCUC

PARP1 and H1.2 stable knockdown HeLa cells were generated using shRNA constructs with the above siRNA sequences cloned into pGPH1/Neo vector (GenePharma, China). shRNAs were transfected with Lipofectamine 2000 and cells were selected with G418 (10 mg/mL) for about 2 weeks. Colonies were picked for cell growth and knockdown efficiencies were verified by immunoblotting.

IR exposure

The biological X-ray irradiator RS2000pro Rad Source was purchased from Rad Source Technologies (Rad Source Technologies, USA) and cells were irradiated with radiation output of 160 KV, 25 mA at a dose rate of 4.125 Gy/min for the indicated dosage.

Nucleic and cytoplasmic fractionation

Cells were suspended in buffer A (10 mM HEPES pH 7.9, 10 mM KCl, 0.1 mM EDTA, 0.1 mM EGTA) supplemented with 1 mM dithiothreitol, 0.15% Nonidet P40 and 1% protease inhibitor cocktail and lysed on ice for 10 min. The supernatant fraction was collected as a cytoplasmic extract after centrifugation at 12,000g for 30 s. The pellet was washed, resuspended in buffer B (20 mM HEPES pH 7.9, 400 mM NaCl, 1 mM EDTA, 1 mM EGTA) supplemented with 1 mM dithiothreitol, 0.5% Nonidet P40 and 1% protease inhibitor cocktail and lysed on ice for 15 min with vigorous vortex every 3 min. The supernatant fraction from the centrifugation was used as the nuclear extract.

***In vitro* degradation assay**

GST-H1.2 or H1.4 was incubated at 37 °C in HEPES buffer (50 mM HEPES pH 7.5, 1 mM DTT, 0.018% SDS) in the presence of 20S proteasomes (Millipore, USA) for the indicated time with or without MG132. The reaction was stopped by adding 5× SDS/PAGE sample buffer and the samples were analyzed by immunoblotting.

Immunoblotting

Equal amounts of proteins were size fractionated by 6-15% SDS-PAGE, transferred onto

nitrocellulose membranes and blocked (5% nonfat milk, 200 mM NaCl, 25 mM Tris·HCl pH 7.5, and 0.05% Tween 20). The membranes were incubated with indicated primary antibodies at 4°C overnight with gentle shaking. Protein bands were detected using a chemiluminescent detection system (Tanon 5200, China) after incubating with secondary antibodies and washing with TBS-T (20 mM Tris, 500 mM NaCl, and 0.1% Tween 20).

Mononucleosome extraction

Cells were harvested into buffer A (10 mM HEPES pH 7.9, 10 mM KCl, 1.5 mM $MgCl_2$, 0.34 M sucrose and 10% glycerol, 1 mM DTT and 0.1% Triton X-100) and lysed on ice for 8 min. Discard the supernatant and dissolve the pellets in buffer B (3 mM EDTA, 0.2 mM EGTA) for 30 min on ice. Discard the supernatant and digest the pellets with MNase at a concentration of 10 U/1 mL in buffer C (10 mM Tris·HCl pH 7.5, 1 mM NaCl, 3 mM $MgCl_2$, 1 mM $CaCl_2$) at 37 °C for 30 min. The supernatant was collected after centrifugation at 12,000 rpm for 10 min and subjected to *in vitro* experiments or immunoblotting.

Mass spectrometry and silver stain

HeLa cells were extracted, immunoprecipitated with anti-IgG or anti-ATM antibodies and subjected to electrophoresis using a 4-12% SDS-PAGE gel. Silver stain was performed using a silver stain kit (Pierce-Thermo Scientific, USA) following the manufacturer's instructions to detect protein bands. Protein bands were cut and sent to PTM BioLabs (Hangzhou, China) for mass spectrometric analysis.

Chromatin immunoprecipitation (ChIP) assay

Briefly, cells were fixed with 1% formaldehyde and lysed in lysis buffer (50 mM Tris·HCl pH 8.0, 5 mM EDTA, 1% SDS). After sonication, the supernatant was collected by centrifugation and pre-cleared in dilution buffer (20 mM Tris·HCl pH 8.0, 2 mM EDTA, 150 mM NaCl, 1% Triton X-100) with protein G or A sepharose and 1 mg/mL salmon sperm DNA. The pre-cleared samples were then incubated with the indicated antibodies and protein G or A sepharose at 4 °C rotating overnight. The beads were then extensively washed and heated at 65 °C for 6 h to reverse the formaldehyde-induced cross-links. The DNA was purified and the DNA expression was analyzed by real-time PCR using the following primers: 5'-TCTTCTTCAAGGACGACGGCAACT-3' (sense), 5'-TTGTAGTTGTACTCCAGCTTGTGC-3' (antisense). The relative occupancy of the indicated protein was calculated by normalizing the occupancy of IgG to 1.

Reverse transcription and real-time PCR

Total RNA was extracted from cells using TRIzol reagent (Invitrogen), following the manufacturer's instructions. Reverse transcription was performed using a HiScript II 1st Strand cDNA Synthesis Kit (Vazyme, China), according to the manufacturer's instructions. The relative expression of target genes was measured on an ABI 7500 (Life Technologies, NY, USA) real-time (RT) PCR system using the SYBR-green dye method. The primers used for RT-PCR were as follows: H1.2: 5'-CCGCCTCTAAAGAGCGTAGC-3' (sense), 5'-AGACCAAGTTTGATACGGCTG-3' (antisense); H1.4: 5'-CTCTGGGGAAGCCAAGCCTA-3' (sense), 5'-TCTTTGGGGTCTTCTTGGCG-3'

(antisense); p21: 5’-AATCCTGGTGATGTCCGACCTGTT-3’ (sense), 5’-AGACCAATCTGCGCTTGGAGTGAT-3’ (antisense); GAPDH: 5’-CAGCAAGAGCACAAGAGGAA-3’ (sense), 5’-CCCCTCTTCAAGGGGTCTAC-3’ (antisense).

Micrococcal nuclease (MNase) sensitivity assay

Cells were harvested into lysis buffer (10 mM HEPES pH 7.9, 10 mM KCl, 1.5 mM $MgCl_2$, 0.34 M sucrose and 10% glycerol, 1 mM DTT and 0.1% Triton X-100). The nuclei were pelleted and digested with MNase (Takara, Japan) at a concentration of 1 U/1 mL in digestion buffer (10 mM Tris·HCl pH 7.5, 1 mM NaCl, 3 mM $MgCl_2$, 1 mM $CaCl_2$) for 1, 2, 5 or 10 min. Genomic DNA was purified by phenol/chloroform extraction and separated by electrophoresis in 1.2% agarose gel. DNA bands were stained by ethidium bromide (EB) and visualized by Gel Doc XR^+ system (Bio-Rad, USA).

Cell cycle synchronization

HeLa cells were treated with thymidine at 2 mM for 18 h and released into drug-free medium for 9 h, after which the cells were again treated with thymidine at 2 mM for 15 h. Cells were then collected at different time after drug withdrawal: immediately, as G1 phase population; 3 h, as S phase population and 9 h as G2/M phase population.
